# ALKBH5/MAP3K8 axis regulates PD-L1+ macrophage infiltration and promotes hepatocellular carcinoma progression

**DOI:** 10.7150/ijbs.70149

**Published:** 2022-08-01

**Authors:** Yu You, Diguang Wen, Lu Zeng, Jiao Lu, Xiao Xiao, Yucheng Chen, Hua Song, Zuojin Liu

**Affiliations:** 1 Hepatobiliary Surgery Department, Second Affiliated Hospital of Chongqing Medical University, Chongqing, 400010, China.; 2 Department of Gastroenterology, the Second Affiliated Hospital of Chongqing Medical University, Chongqing, 400010, China.; 3 Department of Gastroenterology, Chongqing University Central Hospital (Chongqing Emergency Medical Center), Chongqing 400010, China.

**Keywords:** M6A, Hypoxia, Tumor-associated macrophages, Immune microenvironment, PD-L1

## Abstract

Hepatocellular carcinoma is one of the most common malignant tumors.M6A is a novel epigenetic modification that have been emerged as vital regulators for the progression of HCC. However, the regulatory role, clinical significance and the details of the modification, such as the impact on the local tumor environment, remain largely unclear. Our study showed that ALKBH5 was highly expressed in HCC and high ALKBH5 expression predicted a worse prognosis of HCC patients. Prediction of ALKBH5 function by tissue samples and single cell sequencing Gene Set Variation Analysis. Primary CD3 + T lymphocytes and bone marrow-derived macrophages were used to evaluate the effect of ALKBH5 on immune microenvironment. The results indicated that ALKBH5 promote HCC cell proliferation, metastasis and PD-L1+macrophage recruitment. Mechanistically the results showed that ALKBH5 regulates MAP3K8 expression in a m6A dependent manner which mediates the proliferation and metastasis of HCC cells. ALKBH5 also promotes the activation of JNK and ERK pathways through upregulating MAP3K8, thus regulating the expression of IL-8 and promoting macrophage recruitment. Taken together, these data show that ALKBH5 promotes HCC growth, metastasis and macrophage recruitment through ALKBH5/MAP3K8 axis and it may serve as a potential diagnostic marker and target for treatment of HCC patients.

## Introduction

Liver cancer is a major health issue and is sixth in incidence rate and fourth in mortality rate among malignant tumors worldwide 1, 2. Hepatocellular carcinoma (HCC) accounts for approximately 70-80% of liver cancers 3. Viral infection and nonalcoholic fatty liver disease have been considered one of the most common risk factors for HCC 4. Abnormal activation of various signaling pathways, such as β-catenin and MAPK, tumor microenvironment shaping, anomalous autophagy flow, and the formation of premetastatic niches and other pathogenesis pathways are known to be involved in the occurrence and development of HCC 5. Epigenetic changes and gene mutations are key driving factors of these pathological processes 6. Given that HCC has fewer intervenible mutation characteristics than other tumors, such as lung and colon cancers, epigenetics play more important roles in the context of HCC 7. Despite progress in investigating detailed molecular mechanisms, effective treatments for HCC are lacking. Therefore, it is vital to understand the molecular pathogenesis underlying HCC to ensure prevention and proper intervention.

Compared with DNA methylation modification, noncoding RNA modification and other epigenetic modifications, m6A modification is the most common modification of eukaryotic mRNA but has not been fully studied 8. M6A has been shown to play a key role in the progression of a variety of tumors, including hepatocellular carcinoma. Previous studies have explored the functions of a variety of m6A-associated proteins in hepatocellular carcinoma with some contradictory results.

In this study, we focused on the mechanism of AlkB homolog 5 (ALKBH5) in HCC. ALKBH5 is the second m6A demethylated enzyme discovered after fat mass and obesity-associated protein (FTO). ALKBH5 was first reported to promote tumor stem formation in gliomas and subsequently reported to promote tumor progression in breast and colon cancer 9. However, in some tumors, such as bladder cancer and pancreatic cancer, ALKBH5 was reported to inhibit tumor growth. These findings suggest the complexity of the action of ALKBH5. Previous studies have reported that ALKBH5 promotes tumor cell proliferation, migration and invasion in hepatocellular carcinoma 10. However, some studies have shown that ALKBH5 overexpression can suppress HCC malignancy 11. Therefore, the role of ALKBH5 in the pathogenesis of HCC is contradictory, warranting further studies.

For hepatocellular carcinoma cells to colonize organisms, the cells require a suitable microenvironment. Such a tumor microenvironment accumulates metabolites and promotes angiogenesis to provide essential nutrients for liver cancer cell proliferation and invasion. Furthermore, such an environment helps tumor cells escape from the immune system by teaming with a variety of immunosuppressive cells 12. Previous study has reported that ALKBH5 can regulate the formation of immune microenvironment and PD-1 immunotherapy in melanoma, but its specific mechanism is unknown 13. Moreover, studies have found that macrophages play a central role in the tumor microenvironment 14. Tumor cells can recruit and educate a large number of tumor-associated macrophages (TAMs) through cytokine secretion, exosomes and metabolite release 15, 16. Macrophages in the tumor microenvironment have been reported to promote tumor growth. The macrophage surface can express PD-L1, a key T cell apoptosis receptor, which promotes the formation of an immunosuppressive microenvironment 15, 17, 18. Furthermore, considering that the liver has the largest macrophage population in the whole body, Kupffer cells, this study focuses on the relationship between ALKBH5 and PD-L1-related immune mechanism, especially the immune regulation mechanism of macrophages 19.

Herein, we established a hepatoma cell model with stable overexpression or knockdown of ALKBH5. Animal models were also established based on immunocompetent mice and deficient mice. Using these models, we investigated the relationship between ALKBH5 and macrophages and the role of ALKBH5 in the development and immunosuppressive microenvironment of HCC.

## Materials and Methods

### Ethics statement

Human HCC samples were obtained via surgical resection in HCC patients from The Second Affiliated Hospital of Chongqing Medical University. The protocols were reviewed and approved by the Ethics Committee of the Second Affiliated Hospital of Chongqing Medical University, Chongqing, China. All animal experiments were conducted in accordance with the ethical standards of animal care and approved by Animal Care Committee of Chongqing Medical University.

### Cell culture and Hepatocellular carcinoma samples

Cell lines (Huh-7, Hep3b, Sk-hep1, HepG2, H22, and THP-1) were purchased from Procell Life Science & Technology Co., Ltd., China. The cells were incubated at 37 °C in a 5% CO2 incubator with DMEM high glucose medium consisting of 10% fetal bovine serum. Anoxic treatment conditions were 93% N_2_, 5% CO_2_ and 2% O_2_ for 48 hours. Bone marrow derived macrophages (BMDM) were extracted according to the previous article 20. After extraction, macrophage colony stimulating factor (M-CSF) with 50 ng/ml was treated for 7 days for downstream experiments. The identification of BMDM differentiation is based on previous studies 20. Spleen cells were extracted from C57 mice and CD3 positive T lymphocytes were sorted by flow cytometry (**Par. 7**). The co-culture system between CD3 positive T lymphocytes and BMDMs was established by Transwell chamber with 0.4 μm pore size in 6-hole plate with 5*10^5^ cells at a 1:1 ratio (**Par. 7**). *In vivo* experiments, TAMs subsets were classified using fluorescence activated cell sorter (FACS) using anti-cd11b and F4 / 80 antibodies (**Par. 6**).

Human HCC samples were obtained via surgical resection in HCC patients from The Second Affiliated Hospital of Chongqing Medical University. The protocols were reviewed and supported by the Ethics Committee of the Second Affiliated Hospital of Chongqing Medical University, Chongqing, China.

### Gene difference analysis and survival analysis

The online data set is to download count data and use “Limma” package for difference analysis. After count data converting to TPM data, “kmsurv” package is used for survival analysis (**Par 1.**).

### Gene set enrichment analysis (GSEA) and gene set variation analysis (GSVA)

Hepatoma transcriptome data were downloaded from The Cancer Genome Atlas (TCGA) databases. Based on the median expression of ALKBH5, the samples were divided into high and low expression groups. Next, GSEA was conducted using GSVA4.0.1 software, and Kyoto Encyclopedia of Genes and Genomes (KEGG) gene sets were used for analysis (**Par. 6**). The single-cell RNA sequencing dataset GSE146115 was downloaded from the GENE EXPRESSION OMNIBUS (GEO) database. Cell types were classified by Seurat R package. Cell subpopulations were annotated using the cellID R package 21. The hepatocyte population in tumor samples was regarded as hepatoma cells for downstream analysis 22. GSVA next analysis used the GSVA R package for hepatocellular carcinoma cell subsets (**Par. 1**). ALKBH5-positive and ALKBH5-negative cells were used to compare differential pathway analysis.

### Virus transfection and drug treatment

The construction of ALKBH5 and MAP3K8 knockdown and overexpression lentivirus was completed by the gene of genechem Co., Ltd. (Shanghai China). When the cells grow to about 40% of the six well plate, add appropriate concentration of lentivirus suspension and cotransfection reagent, and change the new solution after 12 hours. The stable cells were screened by puromycin with appropriate concentration. The construction of YTHDF2 and HIF-1α siRNA and IL-8 overexpression plasmids was completed by GenePharma Co., Ltd. (Shanghai, China). ALKBH5 mutant H204A was constructed by genechem Co., Ltd. (Shanghai China). Cycloleucine was purchased from rhawn Co., Ltd. (Shanghai China). When the cells grow to about 70% of the six well plate, HCC cells were treated with 0, 20, 80 mM for 12 hours (**Par. 4**).

### Western blot and RT-qPCR

RIPA lysis buffer (Beyotime Biotechnology, Shanghai, China) was used for cell and tissue lysis. Protein was extracted by centrifugation at 12,000 g and detected by BCA assay at 562 nm. A rapid gel preparation kit (Beyotime Biotechnology, Shanghai, China) was used to configure the gel distribution at 130 V constant voltages and 240 mA constant current elec-troconversion. Primary antibody was incubated at 4 °C overnight. Secondary antibody was incubated at 37 °C for 1 hour. Phosphate buffered saline was used to wash test strips three times for five minutes each. High-sensitivity ECL (Bio-Rad, USA) solution was used for the exposure strip. ImageJ software was used to analyze results. RNA extraction and qPCR were performed using RNAiso reagent and a PrimeScript™ Reverse Transcriptase kit (Takara, Shiga, Japan) following manufacturer instructions (**Par. 1-7**).

### Enzyme Linked Immunosorbent Assay (ELISA)

Use the standard sample of ELISA kit to formulate standard curve. Add the sample and incubate it with the coated antibody at 37 °C for 90 minutes, then add SAB for 30 minutes, TMB will develop color, and the OD value will be detected at 450 nm. M6A abundance was detected by colorimetry. After adding 100-300 ngRNA, add coated m6A antibody for binding. After washing with the buffer, add detection antibody and enhancer, and measure the OD value at 450 nm (**Par. 4**).

### Immunohistochemistry (IHC) and immunofluorescence (IF)

IHC was performed using an immunohistochemistry kit (Solarbio, Beijing, China). Sam-ples were prepared through gradient dehydration of paraffin sections and EDTA antigen thermal repair. Primary antibody was incubated ovenight. After three washes with PBS, secondary antibody was incubated at room temperature for 1 hour. Samples were then subjected to DAB staining and hematoxylin staining and measured by immunofluorescence (**Par. 1, 2, 5 and 7**).

### Fluorescence *in situ* hybridization (FISH)

FISH was performed using an mRNA *in situ* hybridization kit (Shanghai GenePharma Co., Ltd., China). Cell samples were first incubated with probe buffer; then, the probes were in-cubated overnight at 37 °C. Labeled cells were imaged by immunofluorescence under a confocal microscope. ImageJ software was used to analyze the colocalization of MAP3K8 mRNA and YTHDF2 protein (**Par. 4**).

### Animal experiments

An immunodeficient mouse model was generated in which Huh-7 or Hep3b **(5×10^6^/100 µl)** was injected subcutaneously into nude mice (**Par. 2 and 5**). T cell-related detection was performed by subcutaneous injection of H22 1×10^6^ cells/100 µl into BALB/C mice (**Par. 7**). Tumor volume was detected every three days, mice were euthanized when tumors grew to suitable volumes, and tumors were completely stripped. All animal experiments were conducted in accordance with the ethical standards of animal care and under the guidance of Animal Care Committee of Chongqing Medical University.

### Cell Counting Kit-8, EDU probe, apoptosis and Cell cycle

A CCK8 test kit was purchased from MCE (MedChemExpress, Shanghai, China). 2000 cells were placed in 96 well plates, cultured for a certain time, diluted with CCK8 solution at 25:1, incubated at 37 °C away from light for 2 hours, and OD450 detection wavelength (**Par. 2 and 5**). EDU probes were performed using an EDU proliferation test kit (Beyotime Biotechnology, Shanghai, China) according to manufacturer instructions (**Par. 2 and 5**). Apoptosis is the centrifugation and resuspension of CD3 positive T cells after treatment, stained with TUNEL kit and tested by flow cytometry (**Par. 7**). Cell cycle assays were conducted based on flow cytometric analysis using a Cell Cycler (Solarbio, Beijing, China) according to manufacturer instructions (**Par. 2 and 5**).

### Migration, invasion and F-actin assay

An 8-μm Transwell chamber (NEST, Jiangsu, China) was used for cell migration tests. Matrix glue (Solarbio, Beijing, China) was applied to the upper layer of the Transwell chamber for invasion assays. Complete DMEM containing 20% fetal bovine serum was added to the lower chamber. 2-5×10^4^ cells (Huh-7, Sk-hep1 and Hep3b) were mixed to 200 ul serum-free medium and added to the upper chamber and waiting for the cells to pass through for 24 hours. Cells were fixed with paraformaldehyde and stained with crystal violet. An F-actin probe was purchased from Beyotime (Beyotime Biotechnology, Shanghai, China). The assays were performed according to manufacturer instructions (**Par. 2 and 5**).

### Measurement of MP3K8 mRNA Stability

When the cells grow to about 70% of the six well plate, stable cells were incubated with actinomycin D for 0 h, 2 h, 4 h, 6 h and 8 h, followed by RT-qPCR. The assays were adapted from Kang et al. 23 (**Par. 4**).

### Flow cytometry

The digested cells were placed in flow buffer (PBS with 0.05%PBS). After incubating in dark for 20 minutes, the cells were washed three times, and the flow cytometry was used to carry out the experiment. FlowJo V10 analysis results (**Par. 3 and 7**).

### Macrophage recruitment assay

THP-1 cells were induced into macrophages after 48 hours by PMA (Sigma, USA) at 100 ng/ml. Macrophage and tumor cell cocultures were performed in Transwell chambers which 5×10^5^THP-1 was placed in the upper chamber, and 5×10 tumor cells were placed in a 24 well plate. The migration time was determined based on the tumor recruitment ability for sub-sequent analysis (**Par. 3 and 6**).

### RNA-seq, m6A-IP-seq and RIP-seq

Total RNA was extracted with RNAiso reagent (Takara, Shiga, Japan). Then, library construction and RNA-seq were performed at Shanghai Yuanshen Biology Co., Ltd. (Shanghai, China), with an Illumina NovaSeq 6000 instrument (Illumina, USA) followed by computational analysis. The criteria for differentially expressed genes were based on a P value < 0.05 and FC > 2 or < -2. The ALKBH5 RIP-seq dataset (GSE144963) and m6A-seq dataset (GSE87515) were analyzed to identify downstream target genes of ALKBH5, for which FC>2 and P<0.05 were considered meaningful (**Par. 4**).

### Statistical analyses

SPSS 24.0 and GraphPad Prism 8.0 (GraphPad, La Jolla, CA) were used for statistical analyses. The measured data are represented as means ± SEM. One-way analysis of variance (ANOVA) or two-tailed Student's t test was conducted to compare quantitative data, whereas nonparametric χ^2^ test was used to analyze qualitative data for which P <0.05 seemed significant (*P < 0.05; **P < 0.01; ***P < 0.001). More detail in supply material.

## Results

### High endogenous ALKBH5 levels correlate with poor outcomes in HCC patients

**Figure 1:**
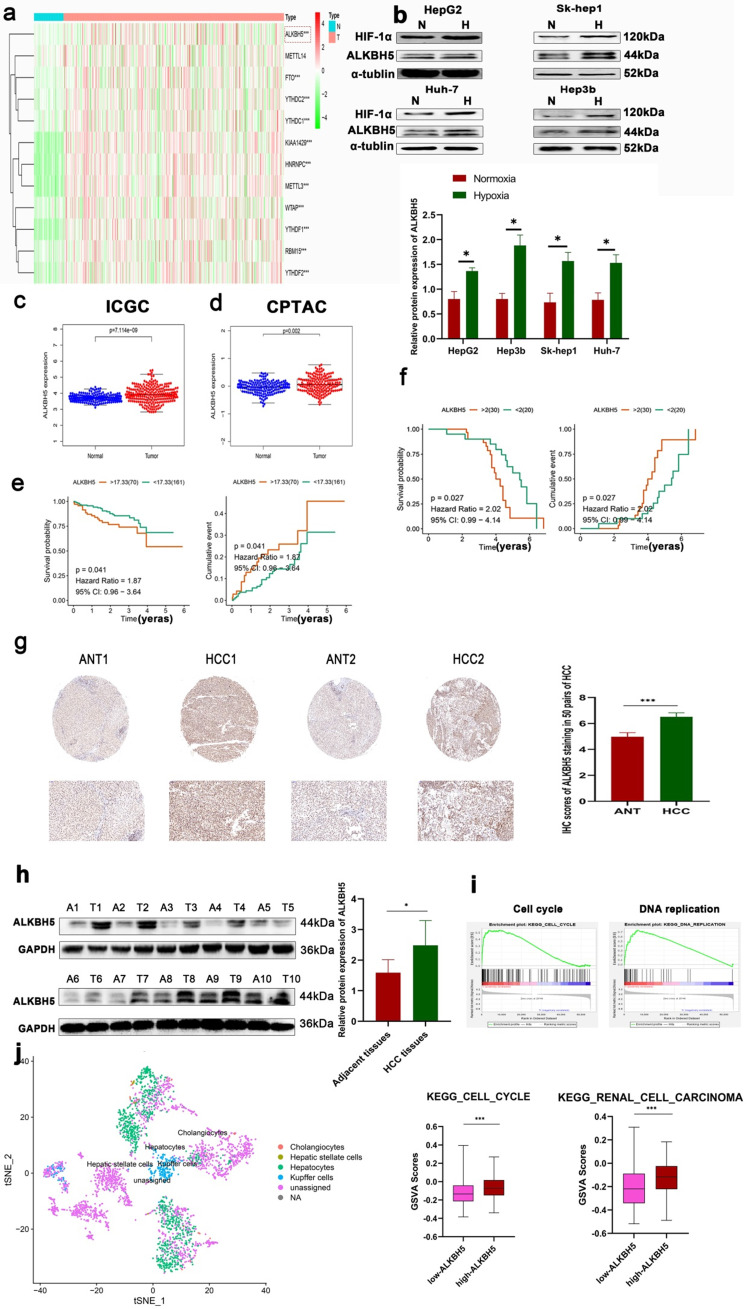
** ALKBH5 is upregulated in patients with HCC and closely related to poor prognosis. (a)** Analysis of differentially expressed m6A-related genes in HCC based on the TCGA database. **(b)** Western blot analysis in HepG2, HUH-7, Sk-hep1 and Hep3b cells with or without hypoxia, n=3. **(c)** Analysis of differentially expressed ALKBH5 in HCC based on the ICGC database. **(d)** Analysis of differentially expressed ALKBH5 in HCC based on the CPTAC database. **(e)** Survival analysis of ALKBH5 in HCC based on the ICGC database. **(f)** Survival analysis of ALKBH5 in HCC based on the 50 pairs IHC tissues. **(g)** IHC assay for ALKBH5 in HCC tissue and adjacent normal tissue n=50. **(h)** Western blot analysis of ALKBH5 in HCC tissue and paracancerous tissue n=10. **(i)** GSEA of ALKBH5 in HCC based on TCGA-LIHC dataset the TCGA database. **(j)** GSVA analysis of ALKBH5 in independent liver tumor cells (Total hepatocytes were regards as HCC cells) based on the single-cell sequencing dataset GSE149614. *P < 0.05; **P < 0.01; ***P < 0.001. Comparisons between the HCC and adjacent tissues were analyzed by paired t test, and comparisons between the other two groups were analyzed by nonpaired t test. Comparisons among multiple groups were analyzed by one-way ANOVA. Cell experiments were repeated in triplicate. All data are presented as the means ± SEM.

In order to understand the expression of m6A-related genes in HCC, we carried out differential gene expression analyses based on the TCGA database, which is an authoritative cancer database widely verified by multiple studies. Similar to previous studies, most m6A-related genes, such as ALKBH5, METTL3 and YTHDF2, were differentially expressed (Fig. 1a). The change of methylase may be an important mechanism in the regulation of tumor progression, but the contrast of the change of methylase between hepatocellular carcinoma and adjacent normal tissue may be caused by a variety of carcinogenic factors or cirrhosis background and another non-tumor factor. Hypoxia is a common phenomenon in solid tumors, accompanied by metastasis, immunosuppression, drug resistance and other malignancy progressions 24. Previous studies and our studies have also found that hypoxia is one of the key regulatory factors of differential gene expression in tumors 25. These results support the importance of m6A in HCC. Based on RT-qPCR, we detected that the expression of the above m6A-related genes changed in the HCC cell line under hypoxia (Fig. S1a). Importantly, only ALKBH5 was upregulated in both HCC and hypoxia which highlights its potential malignant function in liver cancer which results can be supported by Zhong L et al. 23. Given that the important role of hypoxia in tumor development, our research group has long focused on the role of hypoxia in liver cancer, and METTL3 YTHDF2 and METTL14 has been studied more frequently in liver cancer 26. Therefore, we emphasized on the role of ALKBH5 in liver cancer. Furthermore, through Western blot assays, we verified that the protein expression of ALKBH5 was upregulated under hypoxia (Fig. 1b). These results were based at the cell level. At the human liver cancer sample level, we found that ALKBH5 was significantly related to the expression of HIF-1α, a key marker of hypoxia at the protein and mRNA levels, by investigating the Timer and CPATAC databases (Fig. S1b). We also analyzed another transcriptome dataset (ICGC-JP dataset) and two proteome databases (CPTAC and HPA databases). Although the inconsistency in the degree of change may be attributed to the heterogeneity of the database which similar with some previous studies 6, these classic databases all suggest that ALKBH5 is upregulated in liver cancer (Fig. 1c, d and S1c) 27-29.

Survival analysis based on the ICGC database also showed that high expression of ALKBH5 was associated with poor prognoses in HCC patients (Fig. 1e). However, in the TCGA LIHC datasets, the survival analysis of ALKBH5 was insignificant (Results not shown). To furthermore verify the above results, based on our own immunohistochemical data, the results showed that the patients with high expression of ALKBH5 had poor prognosis (Fig. 1f). As shown in Table 1, high expression 45 of ALKBH5 in HCC tissues was significantly correlated with tumor size (P= 0.0014) and TNM stage (P= 0.0157).

To assess the expression levels of ALKBH5 in HCC patients, we performed an IHC assay. The results showed that ALKBH5 was mainly expressed in the nucleus, and its expression was upregulated in HCC (Fig. 1g). Western blot and RT-qPCR assays were performed, which further confirmed these results (Fig. 1h and S1d). To explore the function of ALKBH5 at the total HCC level, we performed GSEA using TCGA-LIHC dataset. The results suggested that the function of ALKBH5 was closely related to the cell cycle and DNA replication (Fig. 1i). Focusing on individual hepatoma cells, we analyzed the single-cell sequencing dataset GSE146115. Similarly, we found that ALKBH5 function was closely related to the cell cycle and renal cell carcinoma (Fig. 1j). Our research group has long focused on the role of macrophages in liver cancer 30. Furthermore, using the TIMER algorithm to evaluate tumor infiltrating cells, we observed that ALKBH5 was significantly associated with macrophage, dendritic cells, B lymphocyte and Neutrophils infiltration which have been reported to be involved in the formation of immunosuppressive microenvironment (Fig. S1e) 31-34. The results showed that ALKBH5 and macrophage are the most related. Although this correlation seems to be a little low, it may be due to the limitations of bioinformatics. In addition, the p value is significant 31. In order to further study the correlation between ALKBH5 and macrophage infiltration, we used clinical samples for immunohistochemistry and found that macrophage infiltration increased significantly in the high expression ALKBH5 group (Fig. S1f). To sum up, these data suggested a potential protumor role of ALKBH5 in HCC.

### ALKBH5 promotes HCC proliferation, invasion and migration

**Figure 2:**
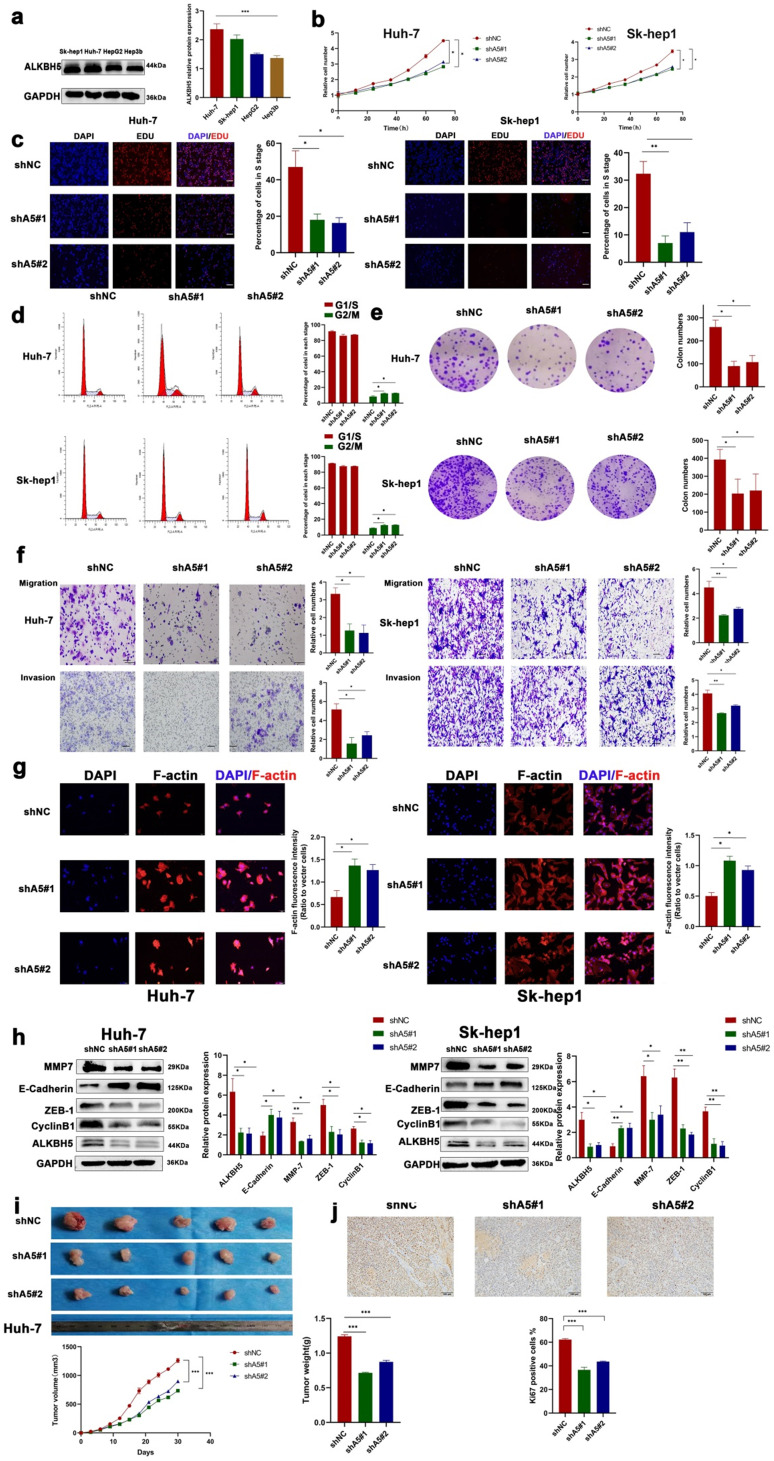
** ALKBH5 promotes the proliferation, migration and invasion of HCC. (a)** Western blot analysis of ALKBH5 protein expression in four HCC cell lines (HepG2, Sk-hep1, HUH-7 and Hep3b n=3. Notes: Proteintech (16837-1-ap) for ALKBH5 antibody **(b)** CCK8 assay for HCC cells with or without silencing ALKBH5 n=3. **(c)** EDU probe for HCC cells with or without silencing ALKBH5 n=3. **(d)** Cell cycle probe showed that cell cycle arrest induced by ALKBH5 silencing in hepatocellular carcinoma cells n=3. **(e)** Colony formation assay for HCC cells with or without silencing ALKBH5 n=3. **(f)** Cell migration and invasion assay for HCC cells n=3. **(g)** F-actin probe for HCC cells observed n=3. **(h)** Western blot was used to analyze the changes of metastasis and proliferation related markers (ZEB-1, MMP-7, CyclinB1 and E-Cadherin) with or without silencing ALKBH5 n=3. **(i)** Subcutaneous tumor in nude mice. Measurement of tumor volume and weight n=5. **(j)** IHC for Ki67 of subcutaneous tumor in nude mice n=5. *P < 0.05; **P < 0.01; ***P < 0.001. All data are presented as the means ± SEM. Student's t-test for independent samples and unequal variances was used to assess statistical significance. Comparisons among multiple groups were analyzed by one-way ANOVA. Comparisons at different time points were analyzed by repeated-measures ANOVA. Cell experiments were independently repeated three times.

To investigate the exact role of ALKBH5 in HCC, we measured the protein expression of ALKBH5 in four HCC cell lines (HepG2, HUH-7, Sk-hep1, and Hep3b) which often used in liver cancer study 35, 36. The results showed that the expression of ALKBH5 was higher in HUH-7 and Sk-hep1 cells and lower in Hep3b cells (Fig. 2a). Next, we stably overexpressed ALKBH5 in Hep3b cells and stably knocked down ALKBH5 in HUH-7 and Sk-hep1 cells with two independent interference sequences (Fig. S2a). The CCK-8 assays indicated that ALKBH5 deficiency inhibited the proliferation ability (Fig. 2b). The EDU probe suggested decreased DNA replication in ALKBH5 knockdown cells (Fig. 2c). Cell cycle analysis showed that cells were arrested in the G2/M phase (Fig. 2d). Additionally, the results of the colony formation assay implied that the long-term proliferative ability of cells was impaired when ALKBH5 was deficient (Fig. 2e). Furthermore, cell migration and invasion were substantially impaired by silencing ALKBH5, as detected by Transwell assays (Fig. 2f). The F-actin fluorescent probe also showed a looser and more divergent pattern of the cytoskeleton after ALKBH5 knockdown (Fig. 2g). Metastasis and proliferation related marker was also downregulated after silencing ALKBH5 (Fig. 2h). The results were reversed when ALKBH5 was over-expressed; the results are shown in Fig. S2b-d.

The *in vivo* role of ALKBH5 was confirmed, and tumor xenograft models were constructed by subcutaneously injecting HCC cells with either stable knockdown (shALKBH5 #1, #2) or overexpression of ALKBH5 (ALKBH5-OE) into nude mice. The depletion of ALKBH5 contributed to reduced tumorigenesis with significantly lower tumor volumes and weights compared with the negative control group (Fig. 2i). The level of Ki67 was downregulated in xenograft tumor tissues with shALKBH5 (Fig. 2j). Moreover, overexpression of ALKBH5 caused an inverse phenotype in xenograft nude mice (Fig. S2e and S2f). We intervened with HIF-1α in two hepatoma cell lines (HUH-7 and Sk-hep1) which significantly inhibited the up regulation of ALKBH5 induced by hypoxia (Fig. S2g and S2h) 37. The above results suggested that ALKBH5 has a tumor malignant behavior-promoting function in HCC and was regulated by hypoxia depended on HIF-1a.

### ALKBH5 recruits PD-L1+ tumor-associated macrophages

**Figure 3:**
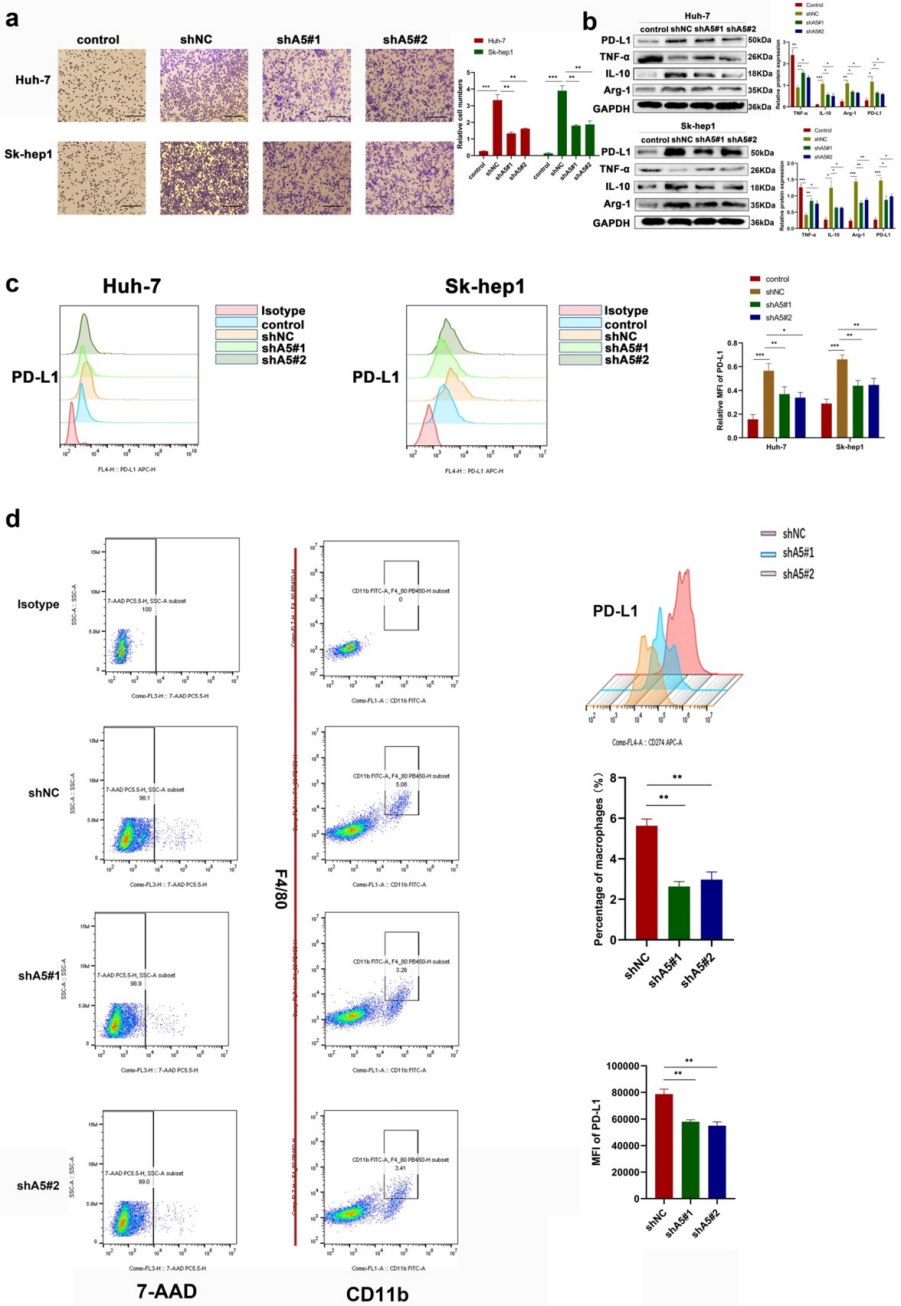
** ALKBH5 promotes the recruitment of PD-L1+ macrophages. (a)** Transwell assays were used to measure the ability of tumor cells with or without silencing ALKBH5 to recruit macrophages. **(b)** RT-qPCR was used to analyze the expression of PD-L1 and polarization markers (Arg-1, IL-10 and TNF-α) in macrophages co-culturing with HCC cells. **(c)** Western blot assay of PD-L1 and polarization markers (Arg-1, IL-10 and TNF-α) expression in macrophages co-culturing with HCC cells. **(c)** Flow probe of PD-L1 expression in macrophages co-culturing with HCC cells. **(d)** Evaluation of PD-L1 positive macrophages in mouse subcutaneous tumors by flow cytometry, F4 / 80 and CD11b double positive labeled macrophages. All data are presented as the means ± SEM. Student's t-test for independent samples and unequal variances was used to assess statistical significance. Comparisons among multiple groups were analyzed by one-way ANOVA. Cell experiments were independently repeated three times.

The effect of ALKBH5 on macrophage recruitment of hepatoma cells was observed. In Transwell *in vitro* experiments, we found that the chemotactic ability of hepatoma cells toward THP-1 cells was decreased after silencing ALKBH5 through coculture (Fig. 3a). PD-L1 is highly expressed in cancer cells and TAMs and has been reported to inhibit T-cell activation and contribute to the tumor immunosuppressive microenvironment 18. Even studies have found that PD-L1 expressed in stromal cells may have clinical significance rather than the tumor itself in some cancers 18. Through bioinformatics analysis, we found that ALKBH5 was positively correlated with PD-L1 expression (Fig. S3a). However, the knockdown of ALKBH5 did not directly affect the expression of PD-L1 in HCC cells (Fig. S3b). Therefore, we determined whether ALKBH5 may regulate the expression of PD-L1 in TAMs. When HCC and THP-1 cells were cocultured, the upregulation of PD-L1 in THP-1 cells induced by HCC cells was impaired after knockdown of ALKBH5; this was confirmed by Western blot and qPCR assays (Fig. 3b and S3c). These results were supported by cellular flow cytometry assays (Fig. 3c). Macrophage polarization is also considered to be one of the important factors for its function. Macrophages in tumor tissue polarize to M2 with high expression of PD-L1. Western blot and qPCR experiment also showed that the silencing of ALKBH5 affected macrophage polarization to M2 through detecting polarized markers (IL-10, TNF-α and Arg-1) (Fig. 3b and S3c). In the above animal experiments, there was lower PD-L1+ tumor-associated macrophage (TAM) infiltration in the ALKBH5 knockdown group than in the control group as shown by flow cytometry (Fig. 3d). After extracting TAMs from mouse-bearing tumor tissue, we found that IL-10 and TGF-β was upregulated and TNF- α was downregulated in the silent ALKBH5 group (Fig. S3d, e).

### MAP3K8 was identified as a potential target of ALKBH5 with an m6A-dependent mechanism in HCC

**Figure 4:**
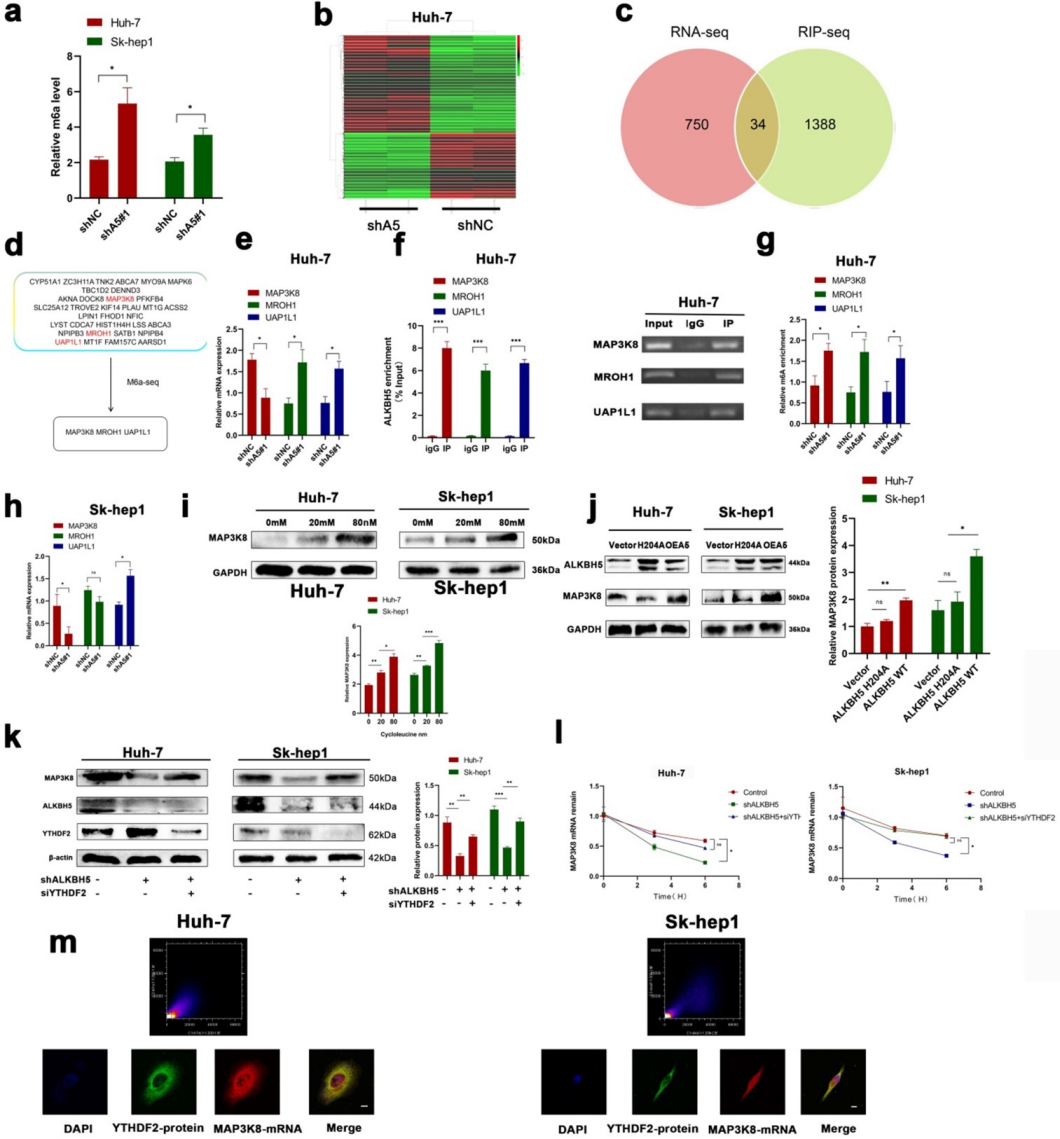
** ALKBH5 regulates MAP3K8 in an m6A -dependent manner. (a)** The overall level of m6A changed after intervention of ALKBH5 expression by m6A colorimetry. **(b)** RNA-seq for HUH-7 cells with or without ALKBH5 knockdown n=2. **(c)** Intersection of the mRNA-seq and RIP-seq datasets (ALKBH5 binding target genes) (GSE144963). **(d)** Intersection of the mRNA-seq and m6A-seq datasets (Genes with increased m6A modification after silencing ALKBH5) (GSE87515). **(e)** RT-qPCR analysis of HUH-7 cells with or without silencing ALKBH5. **(f)** RIP-qPCR analysis of HUH-7 with or without silencing ALKBH5 in order to detect the combination of ALKBH5 with three genes (MAP3K8, MROH1, and UAP1L1). **(g)** M6a-IP-qPCR analysis of HUH-7 with or without silencing ALKBH5. **(h)** RT-qPCR analysis of Sk-hep1 with or without silencing ALKBH5. **(i)** Western blot analysis of the effect of cycloleucine on MAP3K8 expression. **(j)** Western blot analysis of the effect of mut-ALKBH5 H204A on MAP3K8 expression. **(k)** Western blot analysis of the effect of silencing YTHDF2 on MAP3K8 expression. **(l)** RT-qPCR analysis of the effect of silencing YTHDF2 on the half-life of MAP3K8 mRNA. **(m)** Colocalization of the YTHDF2 protein and MAP3K8 mRNA was observed by IF-FISH. *P < 0.05; **P < 0.01; ***P < 0.001. All data are presented as the means ± SEM. Student's t-test for independent samples and unequal variances was used to assess statistical significance. Comparisons among multiple groups were analyzed by one-way ANOVA. Comparisons at different time points were analyzed by repeated-measures ANOVA. Cell experiments were independently repeated three times.

To understand the underlying regulatory mechanism by which ALKBH5 promotes HCC, we measured the overall change in m6A in the knockdown of ALKBH5; the results showed that the overall level of m6A was upregulated (Fig. 4a). mRNA-seq was performed to identify changes in mRNA expression extensively in the ALKBH5 knockdown group. A total of 784 differentially expressed genes were identified (Fig. 4b). To narrow the range, we obtained three genes (MAP3K8, MROH1, and UAP1L1) by crossing the above mRNA-seq data with the online ALKBH5 RIP-seq dataset (GSE144963) and m6A-seq dataset (GSE87515) (Fig. 4c and 4d). In HUH-7 cells, we performed RT-qPCR, RIP-qPCR and m6A-qPCR, which confirmed these results (Fig. 4e-g). However, in SK-hep1 cells, only MAP3K8 and UAP1L1 were successfully verified (Figures S4a, S4b and 4h). We directed our attention to MAP3K8 because our previous study identified MAP3K8 as an immune-related molecule 38. Cycloleucine, a drug that reduces m6A, was added to HUH-7 and Sk-hep1 cells, and the expression of MAP3K8 was upregulated (Fig. 4i) 39, 40. Next, we upregulated the mutant ALKBH5 H204A which a mutant without m6A activity was reported previously; this did not affect the expression of MAP3K8 (Fig. 4j) 13. These results suggested that ALKBH5 regulated MAP3K8 in an m6A manner. In detail, YTHDF2 is the most classic m6A binding protein regulating mRNA degradation. We knocked down YTHDF2 and observed restored protein expression and mRNA attenuation of MAP3K8 (Fig. 4k and 4l). IF-FISH revealed colocalization between YTHDF2 and MAP3K8 (Fig. 4m). RIP-qPCR assays also showed that silencing ALKBH5 promoted the binding of the YTHDF2 protein to MAP3K8 mRNA (Fig. S4c). Thus far, our data showed that ALKBH5 regulated MAP3K8 expression in a YTHDF2-mediated m6A-mediated manner.

### MAP3K8 acts as a tumor promoter and mediates the effects of ALKBH5 in HCC

**Figure 5:**
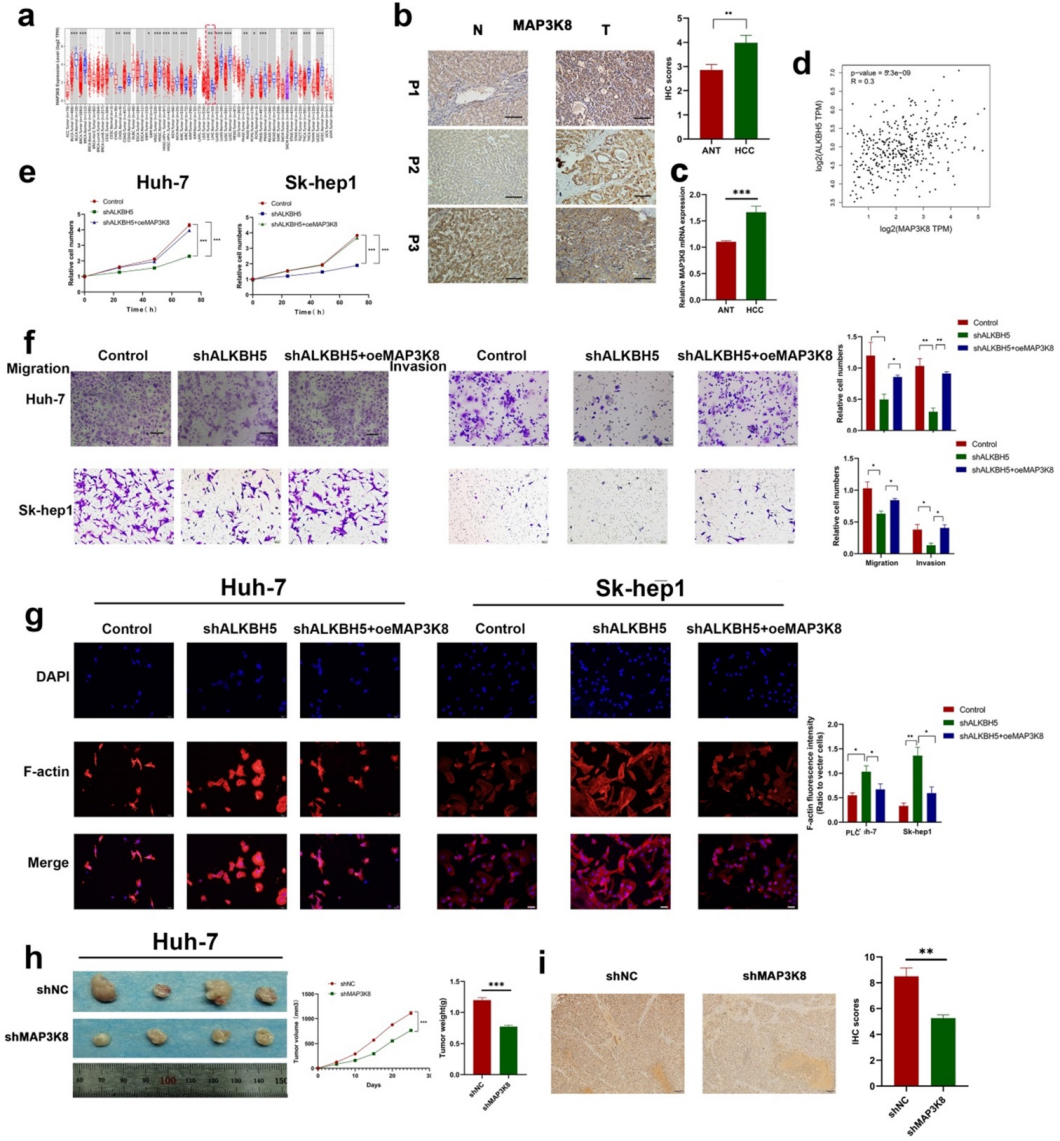
** MAP3K8 mediates ALKBH5 to promote the proliferation, migration and invasion of hepatocellular carcinoma. (a)** Timer database analysis showed that MAP3K8 was highly expressed in HCC. **(b)** Immunohistochemical showed that MAP3K8 was highly expressed in HCC, n=50. **(c)** RT-qPCR analysis showed that MAP3K8 was highly expressed in HCC. n=70 **(d)** Correlation analysis between ALKBH5 and MAP3K8 based on the TCGA-LIHC dataset. **(e)** CCK-8 analysis of the role of MAP3K8 on the proliferation of hepatoma cells. **(f)** Transwell assays were used to analyze the effect of MAP3K8 on the migration and invasion of hepatoma cells. **(g)** Effect of MAP3K8 on the cytoskeleton of HCC detected by the F-actin probe. **(h)** Subcutaneous tumor in nude mice with or without MAP3K8 knockdown in HUH-7 cells. Measurement of tumor volume and weight n=4. **(i)** IHC for Ki67 of subcutaneous tumor in nude mice n=4. *P < 0.05; **P < 0.01; ***P < 0.001. All data are presented as the means ± SEM. Comparisons between the HCC and adjacent tissues were analyzed by paired t test, and comparisons between the other two groups were analyzed by non-paired t test. Comparisons among multiple groups were analyzed by one-way ANOVA. Cell experiments were independently repeated three times.

To further explore the underlying role of MAP3K8 in HCC, we analyzed the Timer database and identified the expression of MAP3K8 in tumor tissues was higher than that in normal tissues; this was further verified by immunohistochemistry and RT-qPCR (Fig. 5a,5b,5c). We also verified the correlation between ALKBH5 and MAP3K8 in clinical HCC samples using the GEPIA datasets (Fig. 5d) 41. Functional validation, including CCK-8 assays, demonstrated that overexpression of MAP3K8 reversed the inhibitory effects of ALKBH5 knockdown on HCC cell growth (Fig. 5e). Transwell and F-actin fluorescent probes confirmed that overexpression of MAP3K8 could rescue migration and invasion after silencing ALKBH5 (Fig. 5f and 5g). Metastasis and proliferation related markers were also reversed by overexpression of MAP3K8 in silencing ALKBH5 (Fig. S5a). *In vivo* experiments further confirmed the above results (Fig. 5h and 5i). The above data confirms that the function of ALKBH5 in HCC is at least partially attributable to MAP3K8. Thus, the inactivation of ALKBH5 may inhibit the progression of HCC via the ALKBH5-MAP3K8 axis.

### MAP3K8-mediated macrophage recruitment capacity of ALKBH5 in HCC is IL-8 dependent

**Figure 6:**
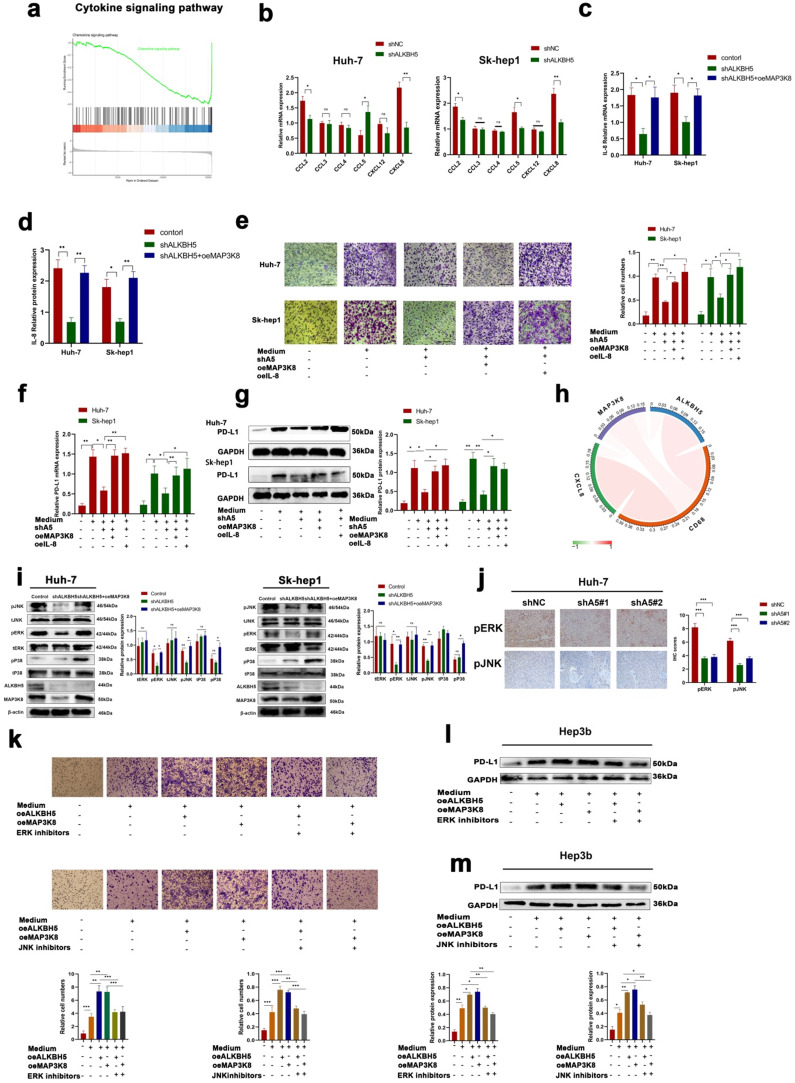
** MAP3K8 mediates the recruitment of PD-L1+ macrophages by ALKBH5. (a)** GSEA of ALKBH5 in HCC based on the above own mRNA-seq data. **(b)** Effect of ALKBH5 on macrophage chemokines measured by RT-qPCR. (c) Effect of MAP3K8 on IL-8 measured by RT-qPCR. **(d)** Effect of MAP3K8 on IL-8 measured by ELISA. **(e)** The effect of MAP3K8 and IL-8 on the recruitment of macrophages was detected by Transwell assay. **(f)** RT-qPCR of PD-L1 expression in macrophages. **(g)** Western blot of PD-L1 in macrophage. **(h)** Correlation analysis of the ALKBH5/MAP3K8/IL-8/CD68 axis based on the ICGC database. **(i)** Western blot analysis of the effect of ALKBH5 and MAP3K8 on ERK/JNK/p38 pathway. **(j)** IHC for p-JNK and p-ERK in subcutaneous tumor. **(l)** Effects of ERK inhibitors (LY3214996) and JNK inhibitors (JNK-IN-8) on the ability of ALKBH5 to interfere with the recruitment of macrophages in hepatoma cells (Hep3b). **(l)** Effects of ERK inhibitors (LY3214996) on the ability of ALKBH5 to induce PD-L1 expression of macrophage in hepatoma cells (Hep3b). **(m)** Effects of JNK inhibitors (JNK-IN-8) on the ability of ALKBH5 to induce PD-L1 expression of macrophage in hepatoma cells (Hep3b). *P < 0.05; **P < 0.01; ***P < 0.001. All data are presented as the means ± SEM. Student's t-test for independent samples and unequal variances was used to assess statistical significance. Comparisons among multiple groups were analyzed by one-way ANOVA. Cell experiments were independently repeated three times.

Previous studies have shown that MAP3K8 regulates the expression of various cytokines in cells. Transcriptome sequencing of intervene ALKBH5 results showed that ALKBH5 was significantly enriched in the regulation of the cytokine pathway (Fig. 6a). Therefore, we speculated that ALKBH5 regulates MAP3K8 thereby mediating the expression of certain cytokines and promoting the recruitment of PD-L1+ TAMs. First, we detected the expression of macrophage chemokines in two hepatoma cell lines (Sk-hep1 and HUH-7). The expressions of IL-8 and CCL2 (MCP-1) were both downregulated, but the IL-8 was most obvious after downregulating ALKBH5 (Fig. 6b). Interestingly, IL-8, a well-known macrophage chemokine, has been shown to be involved in the regulation of PD-L1 expression 42. The overexpression of MAP3K8 reversed the downregulation of IL-8 after silencing ALKBH5 (Fig. 6c and 6d). By analyzing the Timer database, we found that MAP3K8 and IL-8 were significantly correlated with macrophage infiltration (Fig. S6a). We also confirmed the ability of the ALKBH5/MAP3K8/IL-8 axis to regulate PD-L1+ macrophage recruitment (Fig. 6e). The ability of the ALKBH5/MAP3K8/IL-8 axis to regulate PD-L1 expression on macrophages was confirmed by qPCR and Western blot (Fig. 6f, 6g and S6b). Based on the ICGC database, we found significant correlation among the ALKBH5/MAP3K8/IL-8/CD68 axis, which supports the above experimental results at the clinical level (Fig. 6h).

Furthermore, previous studies have reported that MAP3K8 is upstream of JNK, P38, and ERK, which are closely related to HCC 43, 44. By Western-blot detection, we found that ERK and JNK activities decreased, but p38 did not change after silencing ALKBH5, in which overexpression of MAP3K8 restored these signals (Fig. 6i). The above results were verified by immunohistochemistry of subcutaneous tumor in nude mice (Fig. 6j). There is a consensus that JNK and ERK pathways regulated the proliferation and metastasis of hepatocellular carcinoma 45. At the same time, some articles reported that they regulate the expression of IL-8 and the recruitment of macrophages 46, 47. We used JNK and ERK inhibitors and found that they were involved in the regulation of IL-8 expression and PD-L1+ macrophage recruitment by ALKBH5/MAP3K8 axis (Fig. S6c, 6d and 6k-m). These results suggested that ERK/JNK signaling is involved in ALKBH5/MAP3K8 regulation of HCC.

### ALKBH5 promotes an immunosuppressive microenvironment in a mouse model

**Figure 7:**
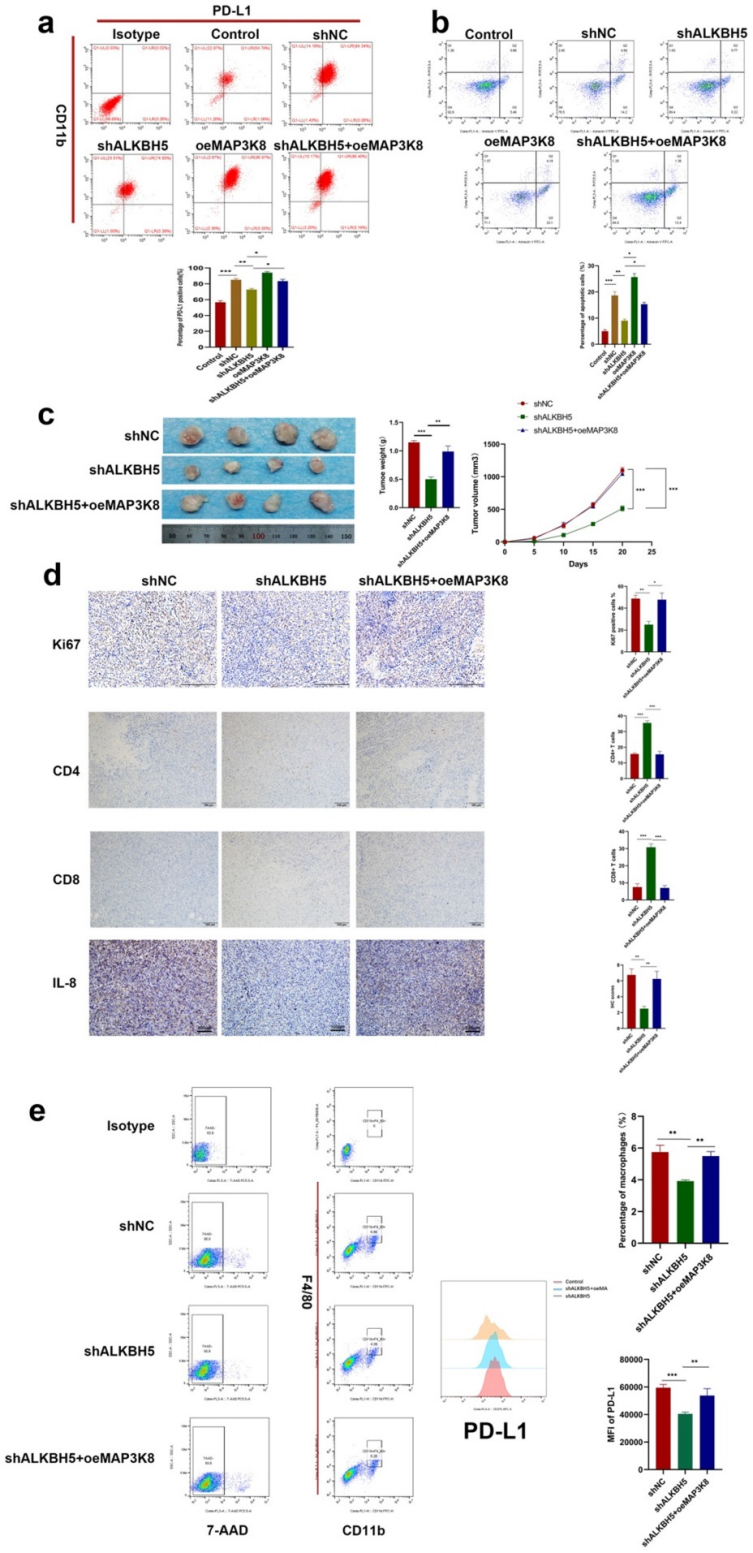
** ALKBH5 promotes immunosuppressive microenvironment. (a)** Effect of MAP3K8 and ALKBH5 on the expression of PD-L1 in macrophages (BMDM) induced by H22 cells by flow cytometry. **(b)** The apoptosis of CD3+T lymphocytes induced by the above treated macrophages (BMDM) was detected by flow cytometry. **(c)** H22 cells stably interfering with ALKBH5 and MAP3K8 were injected subcutaneously into mice, n=4. **(d)** The infiltration of CD4+ and CD8+ T lymphocytes and Ki67 positive percentage was analyzed by IHC n=4. **(e)** The ratio of PD-L1 + macrophages were analyzed by flow cytometry. TAMs were double positive for CD11b and F4 / 80 n=4. *P < 0.05; **P < 0.01; ***P < 0.001. All data are presented as the means ± SEM. Student's t-test for independent samples and unequal variances was used to assess statistical significance. Comparisons among multiple groups were analyzed by one-way ANOVA. Cell experiments were independently repeated three times.

A large number of studies have confirmed the increased infiltration of tumor-associated macrophages and the high expression of PD-L1 are key factors contributing to an immunosuppressive microenvironment 48. To evaluate the effect of ALKBH5 on the tumor immunosuppressive microenvironment, we constructed the mouse hepatoma cell line H22 with stable ALKBH5 knockdown and stable MAP3K8 overexpression. When H22 cells were co cultured with BMDM (bone marrow derived macrophages), silencing ALKBH5 could damage the ability of inducing BMDM to express PD-L1. Overexpression of MAP3K8 could reverse the loss of PD-L1 (Fig. 7a). When the above BMDM was co-cultured with mouse primary spleen CD3 + T lymphocytes, it was also found that the ability of promoting apoptosis of T lymphocytes by BMDM induced by hepatoma cells was weakened after the silencing of ALKBH5 (Fig. 7b, S7a and S7b).

The above hepatoma cells were injected subcutaneously into BALBc mice. Not surprisingly, the tumors in the ALKBH5 knockdown group grew more slowly and had smaller volumes and weights than those in the control group (Fig. 7c). Immunohistochemistry showed that there was lower Ki67 positive cell and more CD4+ and CD8+ T lymphocyte infiltration in the ALKBH5 knockdown group (Fig. 7d). Flow cytometry analysis showed that silencing ALKBH5 resulted in less infiltration of PD-L1 + macrophages, and overexpression of MAP3K8 could reverse its effect (Fig. 7e). These results indicated the role of ALKBH5 in the immunosuppressive microenvironment and also further supported these *in vitro* conclusions.

## Discussion

The pathogenesis of HCC is considered a complex, multifactorial process. Recently, emerging evidence has suggested that abnormal signal pathway activation, tumor microenvironment formation, and immunosuppressive states are involved in the occurrence and development of HCC. Although a few studies have reported ALKBH5 involvement in the pathogenesis of HCC, their results have been contradictory 10, 11, 49. This may be attributed to the selection of tumor cell lines, the efficiency of virus transfection and experimental conditions. Similar contradictory results were reported in studies of other m6A-related proteins in HCC, such as YTHDF2, METTL14 and METTL3, which suggests the complexity of the m6A mechanism in HCC 50-54. Additionally, previous studies only observed the effect of ALKBH5 on tumor cells but did not comprehensively investigate the pertinent underlying mechanisms. Immunity plays an important role in tumor. A consensus is reached that the immune microenvironment has an extremely critical function in the occurrence of liver cancer. Compared with previous studies, we have conducted studies on immune non-defective mice, which has increased the data persuasion. In a study that ALKBH5 inhibited liver cancer, Huh-7 cell line was also used; however, only *in vitro* experiments were carried out with siRNA, and the siRNA may be affected by transfection efficiency and time 11. Besides, Depmap data based on CRISPR data also showed that silencing ALKBH5 impaired the proliferation of hepatoma cells including Huh-7. In two studies supporting ALKBH5 to promote liver cancer, only 2 liver cancer cell lines (HepG2 and MHCC-97H) were used, and only knockdown of ALKBH5 was done 10, 49. We used several authoritative liver cancer data sets and collected clinical samples to verify the high expression of ALKBH5 in liver cancer and its correlation with poor prognosis. Therefore, our data confirmed that ALKBH5 promotes proliferation, invasion and PD-L1+ macrophage recruitment in hepatoma cells. Based on high-throughput sequencing data and experimental verification, MAP3K8 was found to be the downstream target gene of ALKBH5; its stability is regulated by the m6A binding protein YTHDF2. MAP3K8, as a key phosphorylation kinase of MAPK pathway, has been proved to promote ERK, JNK and p38 phosphorylation and signal transduction. Our study novelty found that MAP3K8 can mediate ALKBH5 activation of the classical cancer-promoting pathways ERK and JNK. ALKBH5 regulates the recruitment of PD-L1+ macrophages mediated by IL-8 and other cytokines through MAP3K8. ALKBH5 can promote the formation of an immunosuppressive microenvironment. Our findings revealed in detail the molecular mechanisms by which ALKBH5 regulates HCC progression.

We analyzed TCGA and ICGC, the two most authoritative tumor databases, and found that the mRNA expression of ALKBH5 was upregulated in HCC. The CPTAC and HPA databases also suggested that ALKBH5 protein expression was upregulated in HCC. The above results were consistent with our experimental data. Previous studies have reported that hypoxia induces upregulation of ALKBH5 in HCC but only at the mRNA level. In this study, we confirmed hypoxia induced upregulation of the ALKBH5 protein in hepatoma cells and observed a correlation between ALKBH5 expression and hypoxia at the clinical and animal levels. Recently, single cell sequencing is a new tool widely used in tumor related research, due to its accurate resolution. Therefore, we performed functional analysis of ALKBH5 in hepatoma cells based on single-cell sequencing data. The results supported the ability of ALKBH5 to promote malignant behavior in hepatocellular carcinoma. Previous studies have shown that m6A-associated proteins can shape the peripheral microenvironment of tumor cells and regulate tumor progression. In hepatocellular carcinoma, YTHDF2 has been reported to affect tumor angiogenesis and inhibit tumor progression 54. However, in the HCC background, it is unclear whether ALKBH5 regulates the tumor immune microenvironment.

Using bioinformatics, we predicted the function of ALKBH5 in hepatocellular carcinoma. In addition to overall tumor function prediction, we used single-cell sequencing data to focus on HCC cells, which increases the reliability of the data. Next, we confirmed ALKBH5 regulates the proliferation and invasion of HCC through multiple functional experiments in three cell lines (HUH-7, Sk-hep1, and Hep3b). Therefore, we believe that ALKBH5 plays a crucial role in the progression of HCC. Timer can be used to evaluate the infiltration of immune cells according to transcriptome sequencing data. Based on this, we found that ALKBH5 is closely related to TAMs. It has been confirmed that macrophages in tumor tissue polarize to M2 type and express PD-L1 to exert immunosuppressive effect, in which IL-8 and CCL2 play an important role 55. Moreover, studies have shown that macrophages with high PD-L1 expression enhanced HCC immunosuppression 14. Therefore, we performed experimental studies that confirmed ALKBH5 regulates macrophage recruitment and PD-L1 expression. The results suggested a new mechanism by which ALKBH5 promotes the progression of HCC. Furthermore, in the immune integrity mouse model, we systematically detected T cell-related indexes, which further supported the above conclusion.

Using mRNA-seq combined with online RIP-seq and m6A-IP-seq datasets, we found promising results that MAP3K8 was the downstream target of ALKBH5 in HCC. MAP3K8, also named TPL2, can mediate the MAPK signaling pathway 14. MAP3K8 also acts as an immune-associated molecule and is involved in multiple immune pathways. Previous studies have found that MAP3K8 can promote tumorigenesis and inflammation in HCC; however, the ability of MAP3K8 to recruit macrophages remains unclear. In this study, we reported the expression of MAP3K8 is upregulated in HCC; this is due to the expression of ALKBH5 and is involved in proliferation, invasion and macrophage recruitment of ALKBH5. In addition, it is a consensus that ERK/JNK pathway promotes the proliferation and invasion of liver cancer, and ZEB-1, MMP7, CyclinB1 and E-cadherin are its downstream targets 56, 57. Therefore, we only tested the effect of intervention of ERK/JNK pathway on ALKBH5 regulating PD-L1 + macrophage recruitment. Furthermore, we analyzed the correlation of the ALKBH5/MAP3K8/IL-8/CD68 regulatory axis through the ICGC database, confirming its clinical rationality.

As with other studies, our research has limitations though we attempted to minimize. The exact m6A site has not been found, due to a large number of sites on MAP3K8, but we mutated the ALKBH5 protein to verify that it regulates MAP3K8 expression in an m6A-dependent manner. In addition, we have only confirmed that ALKBH5 is upregulated by hypoxia, but the role of anoxic background still needs further experimental study. Although our study mainly emphasizes the role of ALKBH5 in the immune microenvironment of liver cancer, further research is needed for some contradictions in previous studies.

## Conclusions

**Figure 8:**
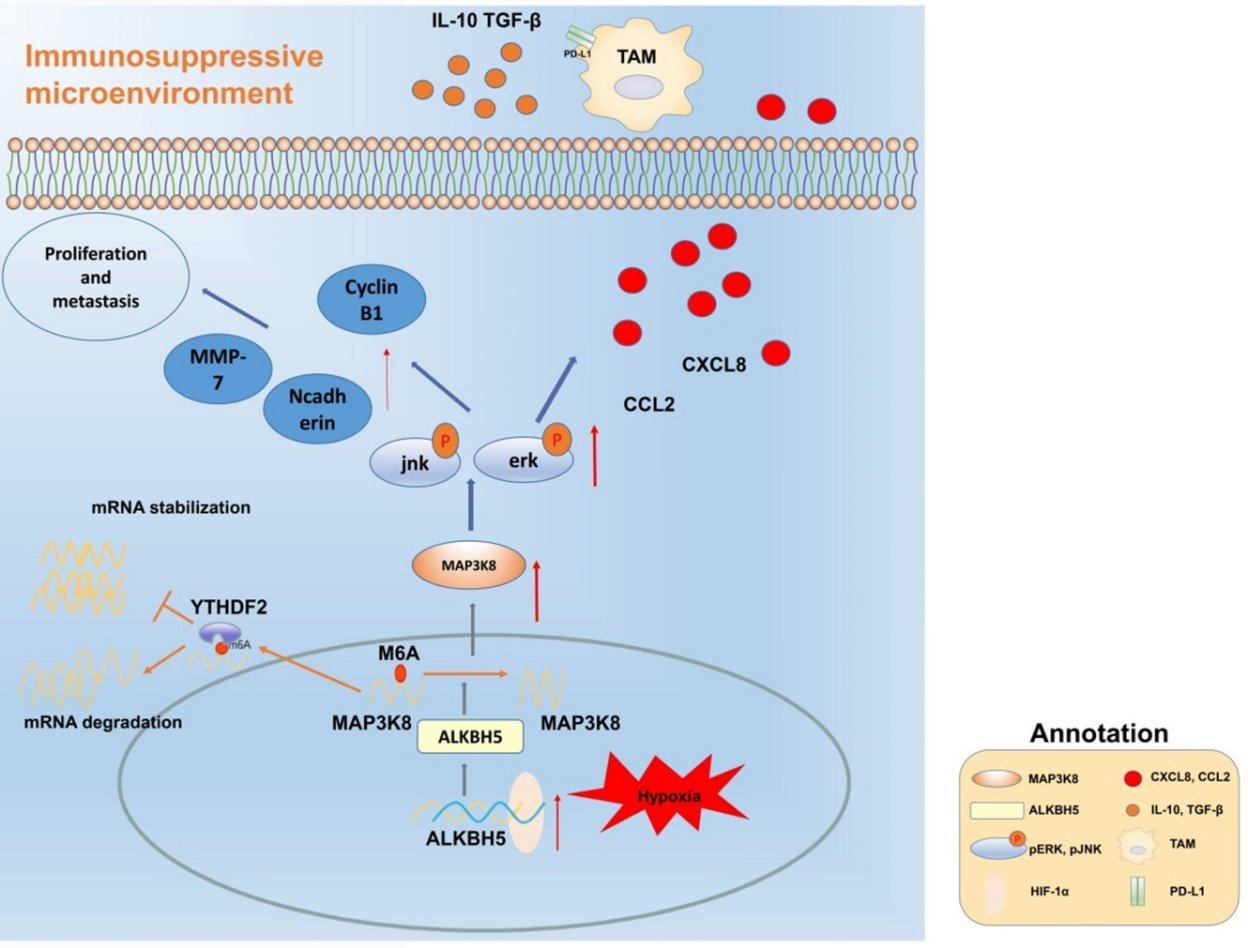
** Mechanism diagram.** ALKBH5 regulates MAP3K8 expression in a m6A dependent manner, promotes the proliferation and metastasis of hepatoma cells, and promotes the expression of IL-8 by activating ERK / JNK pathway, thereby recruiting PD-L1 + macrophages to participate in shaping the immunosuppressive microenvironment.

Our data reveal that ALKBH5 promotes the progression of HCC by regulating the proliferation, invasion and macrophage recruitment of HCC cells. Mechanistically, the ALKBH5/MAP3K8 axis promoted HCC tumorigenesis, metastasis and macrophage recruitment by activating the ERK/JNK pathway and regulating IL-8 expression (Fig. 8). In detail, ALKBH5 regulates the stability of MAP3K8 mRNA through the mechanism of m6A mediated by YTHDF2. However, it is necessary to further explore whether ALKBH5 has other mechanisms in liver cancer or its subtypes in the future. In addition, considering the mechanism we have described, our findings may provide new insights and potential targets into the prognostic or therapeutic treatment of liver cancer.

## Supplementary Material

Supplementary materials and methods, figures.

## Figures and Tables

**Table 1:** Association of ALKBH5 expression with clinicopathological features of 50 HCC patients

Characteristics	ALKBH5 Low (n=25)	Expression High (n=25)	P value
**Age** y≥55 years old	14	16	0.7733
**Gender**	15	18	0.5512
Female			
**Serum AFP** ≥45	14	19	0.2321
**Tumor size** ≥ 5.0 cm	5	17	0.0014
**TNM stage** III/IV	4	13	0.0157
**HBV** Positive	31	40	0.070

## Abbreviations

HCC: Hepatocellular carcinoma
ALKBH5: AlkB homolog 5
M6A: N6-methyladenosine
TAMs: Tumor-associated macrophages
PD-L1: Programmed cell death 1 ligand 1
TCGA: The Cancer Genome Atlas
ICGC: International Cancer Genome Consortium
CPTAC: Clinical Proteomic Tumor Analysis Consortium
TIMER: Tumor Immune Estimation Resource
HPA: Human Protein Atlas
GSEA: Gene Set Enrichment Analysis
GSVA: Gene Set Variation Analysis
MFI: Mean fluorescence intensity
ANT: Adjacent normal tissue
RIP: RNA immunoprecipitation
FISH: Fluorescent *in situ* hybridization
IL-8: Interleukin-8
