# Supplementary material for: ALKBH5/MAP3K8 axis regulates PD-L1+ macrophage infiltration and promotes hepatocellular carcinoma progression
Source: Int J Biol Sci. 2022 Aug 1;18(13):5001–18. doi: 10.7150/ijbs.70149 (PMC9379398; doi:10.7150/ijbs.70149)
Supplement: Supplementary file 1 — Supplementary materials and methods, figures. [file ijbsv18p5001s1.pdf]

Supplementary Materials and Methods

Details of specific use of antibodies

Antibody name	Company	Application
Gapdh (60004-1-Ig)	Proteintech	WB(1:1000)
β-actin (60008-1-Ig)	Proteintech	WB(1:1000)
α-tubulin (11224-1-AP)	Proteintech	WB(1:4000)
YTHDF2 (24744-1-AP)	Proteintech	WB(1:5)/IF(1:100)/IP(1:50)
ALKBH5 (16837-1-AP)	Proteintec	IP(1:100)/WB(
Arg-1 (ab233548)	h	1:1000)
TNF-α (ab183218)	Abcam	WB(1:1000)
IL-10 (ab133575)	Abcam	WB(1:1000)
ZEB-1 (21544-1-AP)	Abcam	WB(1:1000)
CDH1 (20874-1-AP)	Proteintec	WB(1:1000)
MMP7 (10374-2-AP)	h	WB(1:1000)
	Proteintech	
	Proteintec	

	h	
Cd274 (66248-1-Ig)	Proteintech	IF(1:200)
MAP3K8 (GTX102711)	Genetex	IHC(1:200)
pERK (4370)	CST	IHC(1:200)/WB(1:2000)
tERK(4695)	CST	WB(1:1000)
pJNK (9255)	CST	IHC(1:200)/WB(1:2000)
tJNK (9252)	CST	WB(1:1000)
pP38 (4511)	CST	WB(1:1000)/IHC(1:200)
tP38 (8690)	CST	WB(1:1000)
Cd274 (13684)	CST	WB(1:1000)
HRP anti-Rabbit (BA1054)	BOSTER	WB(1:8000)
HRP anti-Mouse (BA1050)	BOSTER	WB(1:8000)
Hif-1α (BF8002)	Affinity	IF(1:100)/WB(1:500)
Ki-67 (AF0198)	Affinity	IHC(1:200)
Anti-mouse F4/80	Biolegend	FC
ALKBH5 (ab195377)	Abcam	IF(1:300)/IHC(1:300)/WB(1:

		1000)
CD4 (ab183685)	Abcam	IHC(1:200)
CD8 (ab217344)	Abcam	IHC(1:200)
MAP3K8 (ab52613)	Abcam	WB(1:1000)
Alexa Fluor® 555 (ab150078)	Abcam	IF(1:400)
Alexa Fluor® 488 (ab150113)	Abcam	IF(1:400)
FITC anti-mouse/human CD11b (101206)	Biolegend	FC
APC anti-mouse CD274 (124311)	Biolegend	FC
PE Rat IgG2a (400507)	Biolegend	FC
APC Rat IgG2b (400611)	Biolegend	FC
Human IL-8 ELISA Kit (EK0413)	BOSTER	ELISA
APC anti-human CD274(329708)	Biolegend	FC
PE anti-mouse CD3 Antibody(100205)	Biolegend	FC
Brilliant Violet 421™ anti-mouse CD8a(100753)	Biolegend	FC
APC anti-mouse CD4(100411)	Biolegend	FC
APC Mouse IgG2b	Biolegend	FC
Alexa Fluor® 488 Rat IgG2a, κ Isotype Ctrl	Biolegend	FC
Cyclin B1	CST	WB(1:1000)

RT-qPCR primer

Gene Name	Forward Primer	Reverse Primer

β -actin (Human)	GTTGAGAACCGTGTACCATGT	TTCCCACAATTTGGCAAGAGC
METTL3 (Human)	CATTGCCCACTGATGCTGTG	AGGCTTTCTACCCCATCTTGA
Mettl14 (Human)	GAACACAGAGCTTAAATCCCCA	TGTCAGCTAAACCTACATCCCTG
FTO (Human)	GCTGCTTATTTCGGGACCTG	AGCCTGGATTACCAATGAGGA
HNRNPC (Human)	CCTTACCATCAAACACGATGGC	ACTTCGAAAAGATTGCCTCCACA
Rbm15 (Human)	ACGACCCGCAACAATGAAG	GGAAGTCGAGTCCTCACCAC
WTAP (Human)	ACTGGCCTAAGAGAGTCTGAAG	GTTGCTAGTCGCATTACAAGGA
YTHDC1 (Human)	GAGGGCCAAATCTCCTACGC	GTCTCATGGTCAGAGCCATATTC
YTHDF1 (Human)	ACCTGTCCAGCTATTACCCG	TGGTGAGGTATGGAATCGGAG
YTHDC2 (Human)	GGTATCCCCTGCCGTATATTTTG	CTTTCCCGTCTCTCTGCGG
KIAA1429 (Human)	AAGTGCCCCTGTTTTCGATAG	ACCAGACCATCAGTATTCACCT
MAP3K8	GAGCGTTCTAAGTCTCTGCTG	GCAAGCAAATCCTCCACAGTTC

(Human)		
CCL2	CATCTCCTACACCCCACGAAG	GGGTTGGCACAGAAACGTC
(Human)		
CCL3	ACTTTGAGACGAGCAGCCAGTG	TTTCTGGACCCACTCCTCACTG
(Human)		
CCL4	GCTTCCTCGCAACTTTGTGGTAG	GGTCATACACGTACTCCTGGAC
(Human)		
CCL5	CCTGCTGCTTTGCCTACATTGC	ACACACTTGGCGGTTCTTTCGG
(Human)		
CXCL12	CTCAACACTCCAAACTGTGCCC	CTCCAGGTACTCCTGAATCCAC
(Human)		
CXCL8	GAGAGTGATTGAGAGTGGACCAC	CACAACCCTCTGCACCCAGTTT
(Human)	GGTGGTAACCTTCAGGCTTCT	CAGCTCGTGGCTGTAAGGAA
Arg-1		
(Human)		
GAPDH	CATCACTGCCACCCAGAAGACTG	ATGCCAGTGAGCTTCCCGTTCAG
(Mouse)		
ALKBH5	TCGGAACCTGTGCTTTCTCTGC	CTTCCTGAGAATGATGACCGCC
(Mouse)		
MROH1	GCCAAGGTAGAGTCAGACATCC	ACACTCTGGACAAGGCACAGCT
(Human)		
UAP1L1	TGGTGGAGTACAGCGAGATCAG	GTGTGGCTTCAGCAAAGGCTCA

(Human)	TTCTCGAACCCCGAGTGACA<br>GACTTTAAGGGTTACCTGGGTTG	CTAAGGCCTGTGCTGTTCCT
TNF- α	CGGCGAAGGCTACACTTACG	TCACATGCGCCTTGATGTCTG
(Human)		CCACCAGCTTTTGGATCACCA
IL-10		
（Human）		
ALKBH5		
(Human)		

ShRNA and siRNA sequence

ID	sequence
shA5#1	CCTCAGGAAGACAAGATTA
(Human)	
ShA5#2	AGAAGGGCCTGTACAACGA
(Human)	
shALKBH5	CCGGCCTTGCTTTGTTGACCATTAACTCGAGTTAATGGTCAACAAAGCAAGGTTTTTTG
(Mouse)	
siYTHDF2	GAACGUCAAGGUCGUGGGAAA
shMAP3K8	GCACTGGAAGTGACAATAAAG
(Human)	
SiHIF-1α	CGAGGAAGAACUAUGAACATT（Sense）; UGUUCAUAGUUCUUCCUCGTT（Antisense）

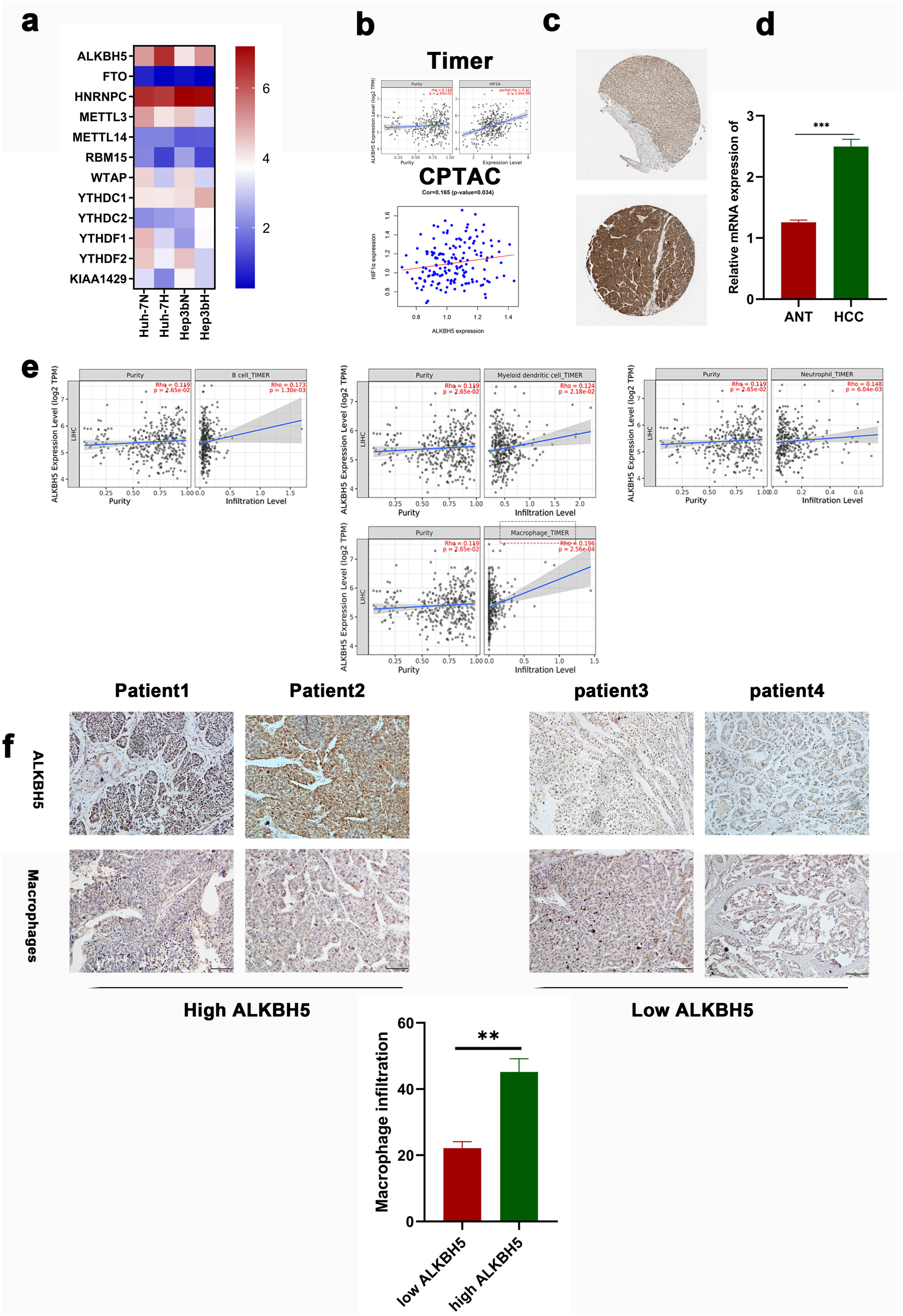
a
ALKBH5
FTO
HNRNPC
METTL3
METTL14
RBM15
WTAP
YTHDC1
YTHDC2
YTHDF1
YTHDF2
KIAA1429
Huh-7N
Huh-7H
Hep3bN
Hep3bH
6
4
2
b
Timer
CPTAC
Cor=0.165 (p-value=0.034)
ALKBH5 expression
HIF1α expression
c
d
Relative mRNA expression of
***
ANT
HCC
e
Purity
B cell_TIMER
Myeloid dendritic cell_TIMER
Neutrophil_TIMER
Macrophage_TIMER
Infiltration Level
ALKBH5 Expression Level (log2 TPM)
LIHC
Rho = 0.119
p = 2.65e-02
Rho = 0.173
p = 1.30e-03
Rho = 0.124
p = 2.18e-02
Rho = 0.148
p = 6.04e-03
Rho = 0.196
p = 2.56e-04
Patient1
Patient2
patient3
patient4
f
ALKBH5
Macrophages
High ALKBH5
Low ALKBH5
Macrophage infiltration
**
low ALKBH5
high ALKBH5

FigureS1

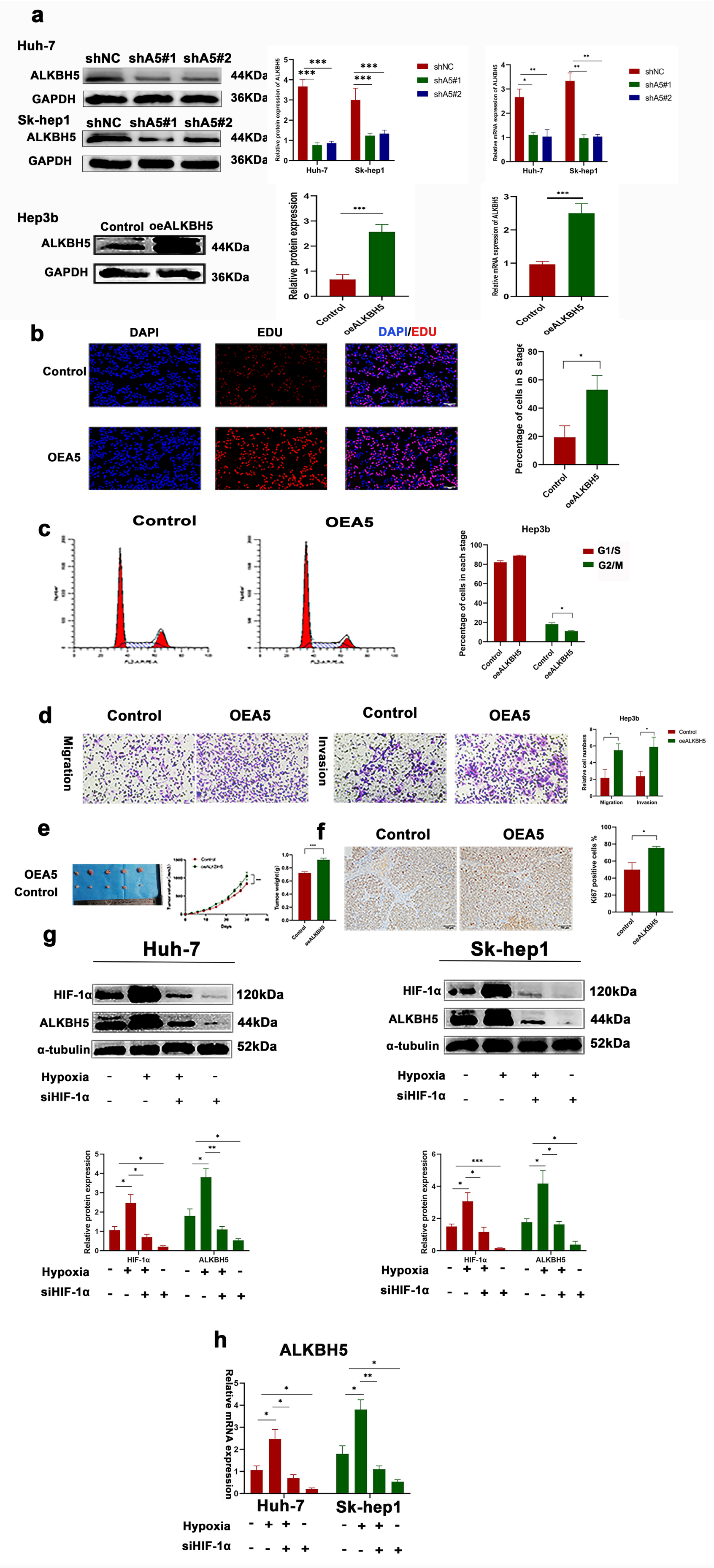
a
Huh-7
shNC shA5#1 shA5#2
ALKBH5 44KDa
GAPDH 36KDa
Sk-hep1
shNC shA5#1 shA5#2
ALKBH5 44KDa
GAPDH 36KDa
shNC
shA5#1
shA5#2
Huh-7
Sk-hep1
Hep3b
Control oeALKBH5
ALKBH5 44KDa
GAPDH 36KDa
Relative protein expression
Control
oeALKBH5
b
DAPI
EDU
DAPI/EDU
Control
OEA5
Percentage of cells in S stage
c
Control
OEA5
Hep3b
G1/S
G2/M
Percentage of cells in each stage
d
Control
OEA5
Migration
Invasion
e
OEA5
Control
f
Control
OEA5
Ki67 positive cells %
g
Huh-7
Sk-hep1
HIF-1α 120kDa
ALKBH5 44kDa
α-tubulin 52kDa
Hypoxia
siHIF-1α
Relative protein expression
h
ALKBH5
Relative mRNA expression
Huh-7
Sk-hep1
Hypoxia
siHIF-1α

FigureS2

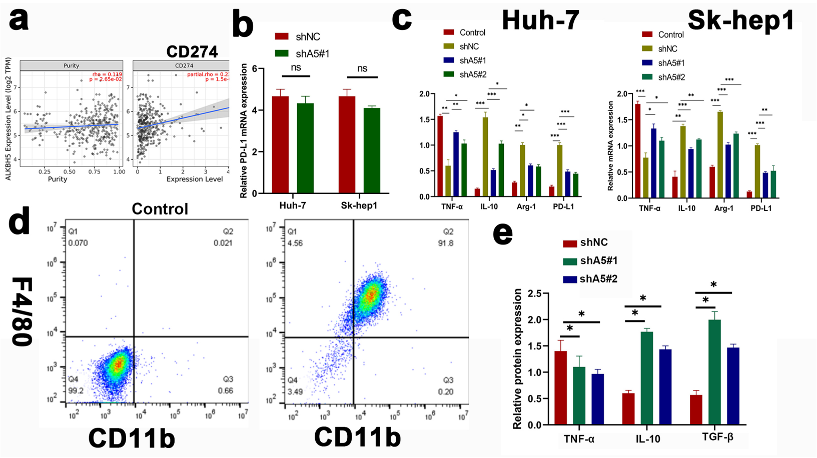

FigureS3

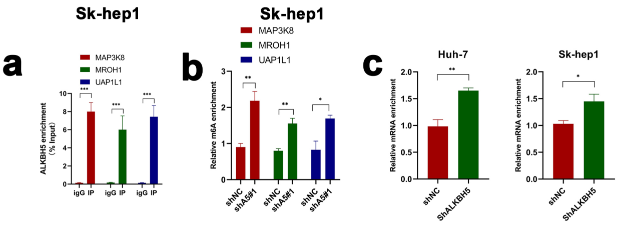

FigureS4

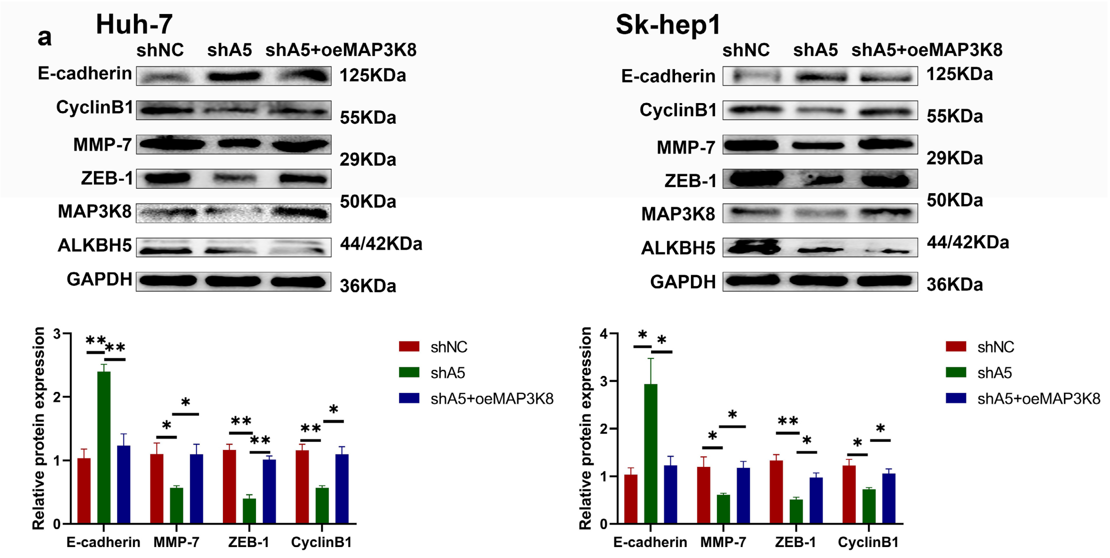

FigureS5

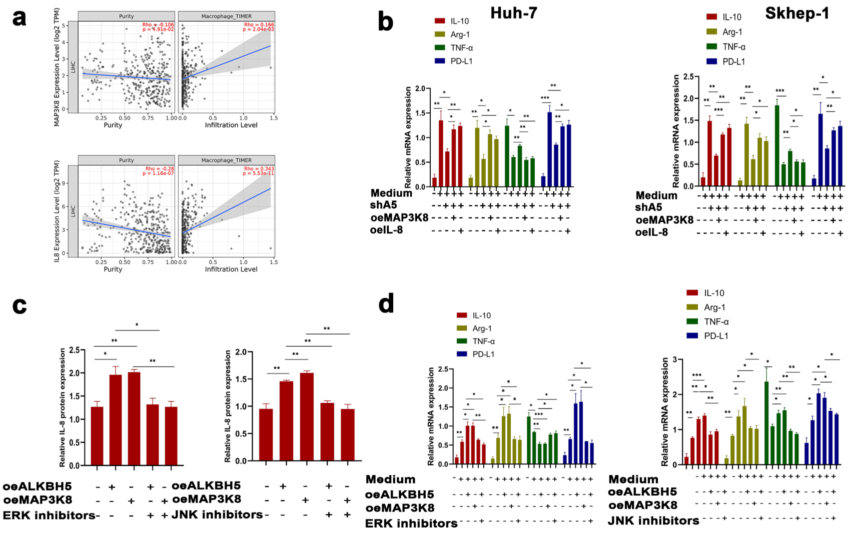

FigureS6

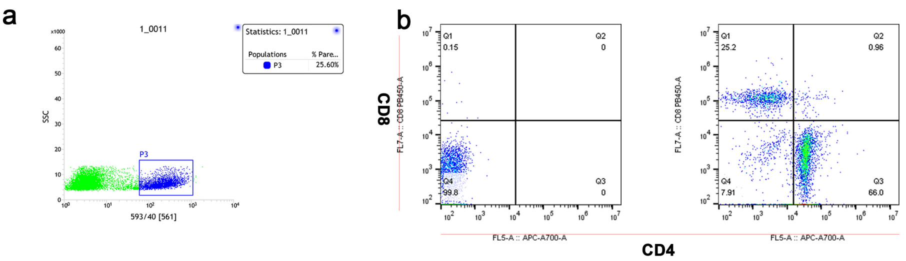

FigureS7

Figure legends

Figure S1. ALKBH5 is upregulated in patients with HCC and closely related to poor prognosis. (a) Detection of m6A related gene expression in hepatocellular carcinoma cells under hypoxia vs normoxia for 72 hours by RT-qPCR. (b) The correlation between ALKBH5 and HIF -1α mRNA and protein expression was based on TIMER (up) and CPTAC (bottom) database. (c) The expression of ALKBH5 was detected in HCC and adjacent tissues based on HPA database. (d) RT-qPCR analysis of ALKBH5 expression in HCC tissue and adjacent normal tissue (n=70). (e) Correlation analysis of ALKBH5 with B lymphocytes, DC cells, macrophages and neutrophils was based on timer database. (f) Based on immunohistochemical data, the difference of TAMs infiltration between high and low ALKBH5 groups was analyzed, CD68 antibody was used to label macrophages, n=50. *P < 0.05; **P < 0.01; ***P < 0.001. All data are presented as the means ± SEM. two groups were analyzed by nonpaired t test. Comparisons among multiple groups were analyzed by one-way ANOVA.

Comparisons at different time points were analyzed by repeated-measures ANOVA. Cell experiments were independently repeated three times.

Figure S2. Functional analysis of over-expression ALKBH5 vs control in Hep3b. (a) Overexpression of ALKBH5 in Hep3b cells. Silencing ALKBH5 in Huh-7 and Sk-hep1. (b) Edu probe for Hep3b. (c) Cell cycle for Hep3b. (d) Cell migration and invasion assay for Hep3b (scale bar, 200μm). (e) Subcutaneous tumor in nude mice. Measurement of tumor volume and weight. (f) IHC for ki67 of subcutaneous tumor in nude mice (scale bar, 100μm). (g) Western blot analysis of ALKBH5 expression after HIF-1 intervention in hepatoma cells (Huh-7 and Sk-hep1). (h) RT-qPCR assay of ALKBH5 expression after HIF-1 intervention in hepatoma cells (Huh-7 and Sk-hep1). *P < 0.05; **P < 0.01; ***P < 0.001. All data are presented as the means ± SEM. Student's t-test for independent samples and unequal variances was used to assess statistical significance. Comparisons among multiple groups were analyzed by one-way ANOVA. Comparisons at different time points were analyzed by repeated-measures ANOVA. Cell experiments were independently repeated three times.

Figure S3. ALKBH5 promotes the recruitment of PDL-1+ macrophages. (a) The correlation between ALKBH5 and PD-L1 (CD274) was analyzed using TIMER database. (b) Effect of silencing ALKBH5 on PD-L1 expression in hepatoma cells. (c) RT-qPCR probe for polarization markers and expression of PD-L1 of THP-1 co-culture with Huh-7/Sk-hep1 (n=3). (d) Flow sorting TAMs, CD11b and F4 / 80 double labeling to verify the screening efficiency. (e) The expression of polarization related cytokines (M1:TNF-α， M2：IL-10；TGF-β) in sorted TAMs was detected by ELISA, n=3. *P < 0.05; **P < 0.01; ***P < 0.001. All data are presented as the means ± SEM. Student's t-test for independent samples and unequal variances was used to assess statistical significance. Comparisons among multiple groups were analyzed by one-way ANOVA. Cell experiments were independently repeated three times.

Figure S4. ALKBH5 regulates MAP3K8 in an m6A -dependent manner. (a) RIP-qPCR analysis of Sk-hep1. (b) M6a-ip-qPCR analysis of Sk-hep1. (c) Effect of ALKBH5 on the binding of YTHDF2 to MAP3K8 by RIP-qPCR. *P < 0.05; **P < 0.01; ***P < 0.001. All data are presented as the means ± SEM. Student's t-test for independent samples and unequal variances was used to assess statistical significance. Comparisons among multiple groups were analyzed by one-way ANOVA. Cell experiments were independently repeated three times.

Figure S5. MAP3K8 mediates ALKBH5 to promote the proliferation, migration and invasion of hepatocellular carcinoma. (a) Western blot was used to analyze the changes of metastasis and proliferation related markers (ZEB-1, MMP-7, CyclinB1 and E-Cadherin) n=3. All data are presented as the means ± SEM. Comparisons among multiple groups were analyzed by one-way ANOVA. Cell experiments were independently repeated three times.

Figure S6. MAP3K8 mediates the recruitment of PDL-1+ macrophages by ALKBH5. (a) Correlation of MAP3K8 (up), IL-8 (bottom) and macrophage infiltration in hepatocellular carcinoma based on TIMER database. (b) RT-qPCR analysis of the effect of HCC cells on macrophage polarization markers and PD-L1. (c) ELISA probe analysis for the effects of ERK/JNK inhibitor on the IL-8 expression in Hep3b. (d) RT-qPCR probe analysis for the effects of ERK/JNK inhibitor on the PD-L1 expression in THP-1. *$P < 0.05$; **$P < 0.01$; ***$P < 0.001$. All data are presented as the means ± SEM. Student's t-test for independent samples and unequal variances was used to assess statistical significance. Comparisons among multiple groups were analyzed by one-way ANOVA. Cell experiments were independently repeated three times.

Figure S7. Identification of T lymphocytes from mouse spleen. (a) Flow cytometric sorting of CD3 positive T lymphocytes. (b) Identification of lymphocyte purity by CD4 and CD8. Cell experiments were independently repeated three times.
